# Cranial Anatomy of *Wendiceratops pinhornensis* gen. et sp. nov., a Centrosaurine Ceratopsid (Dinosauria: Ornithischia) from the Oldman Formation (Campanian), Alberta, Canada, and the Evolution of Ceratopsid Nasal Ornamentation

**DOI:** 10.1371/journal.pone.0130007

**Published:** 2015-07-08

**Authors:** David C. Evans, Michael J. Ryan

**Affiliations:** 1 Department of Natural History, Royal Ontario Museum, 100 Queen’s Park, Toronto, Ontario, M5S 2C6 Canada; 2 Department of Ecology and Evolutionary Biology, University of Toronto, 25 Willcocks Street, Toronto, Ontario, M5S 3B2 Canada; 3 Department of Vertebrate Paleontology, Cleveland Museum of Natural History, 1 Wade Oval Drive, University Circle, Cleveland, Ohio 44106, United States of America; University of Pennsylvania, UNITED STATES

## Abstract

The fossil record of ceratopsid dinosaurs between the occurrence of their proximate sister taxa in the Turonian and the beginning of their well-documented radiation from the late Campanian of North America onwards (approximately 90 and 77 Ma) is poor, with only seven taxa described from this early period in their evolution. We describe a new taxon of a highly adorned basal centrosaurine, *Wendiceratops pinhornensis* gen. et sp. nov., from the lower part of the Oldman Formation (middle Campanian, approximately 78-79 Ma), Alberta, Canada. Over 200 bones derived from virtually all parts of the skeleton, including multiple well-preserved specimens of the diagnostic parietosquamosal frill, were collected from a medium-density monodominant bonebed, making the new taxon one of the best-represented early ceratopsids. The new taxon is apomorphic in having epiparietals at loci 2 and 3 developed as broad-based, pachyostotic processes that are strongly procurved anterodorsally to overhang the posterior and lateral parietal rami, and an ischium with a broad, rectangular distal terminus. Although the morphology of the nasal is incompletely known, *Wendiceratops* is inferred to have a large, upright nasal horn located close to the orbits, which represents the oldest occurrence of this feature in Ceratopsia. Given the phylogenetic position of the new taxon within Centrosaurinae, a enlarged nasal horn is hypothesized to have arisen independently at least twice in ceratopsid evolution.

## Introduction

Ceratopsid dinosaurs are common components of the latest Cretaceous (Campanian-Maastrichtian) terrestrial faunal assemblages of North America [1, 2], where their remains often constitute over 20% of the dinosaur skeletal material recovered in terms of relative abundance [3, 4]. Ceratopsidae consists of two diverse clades recognized at the subfamily level: Centrosaurinae and Chasmosaurinae [1]. Members of the two subfamilies are distinguished primarily by their cranial ornamentation. Centrosaurines are characterized by short parietosquamosal frills with short squamosals and typically ornate parietals, while chasmosaurines have longer frills with elongate, triangular-shaped squamosals and relatively simple epimarginal ornamentation [1].

Recent discoveries from the Belly River Group of southern Alberta [6–8] and the Wahweap and Kaiparowits formations of Utah [2, 9–11] have revealed a remarkable diversity of Centrosaurinae from both northern and southern Laramidia [2]. The recent descriptions of new taxa from early to middle Campanian strata have shown that early centrosaurines also had large brow horns similar to those of many chasmosaurines, and relatively unadorned frills [6, 11]. However, the fossil record of the early radiation of ceratopsids between the occurrence of their proximate sister taxa in the Turonian and their well-documented radiation from the Late Campanian of North America onwards remains poor [2]. Only seven ceratopsid species from this period in their evolution (between 90 and 77 Ma) have been named to date (*Albertaceratops nesmoi* [6], *Avaceratops lammersi* [12, 13], *Coronosaurus brinkmani* [8], *Diabloceratops eatoni* [9], *Xenoceratops foremostensis* [7], *Medusaceratops lokii* [14], and *Judiceratops tigris* [15]). Many of these taxa are represented by limited fossil material and extremely fragmentary specimens (e.g., *Xenoceratops*, *Judiceratops*, *Medusaceratops*), making knowledge of the origin and early evolution of the characteristic ceratopsid cranial ornamentation incomplete.

**Fig 1:**
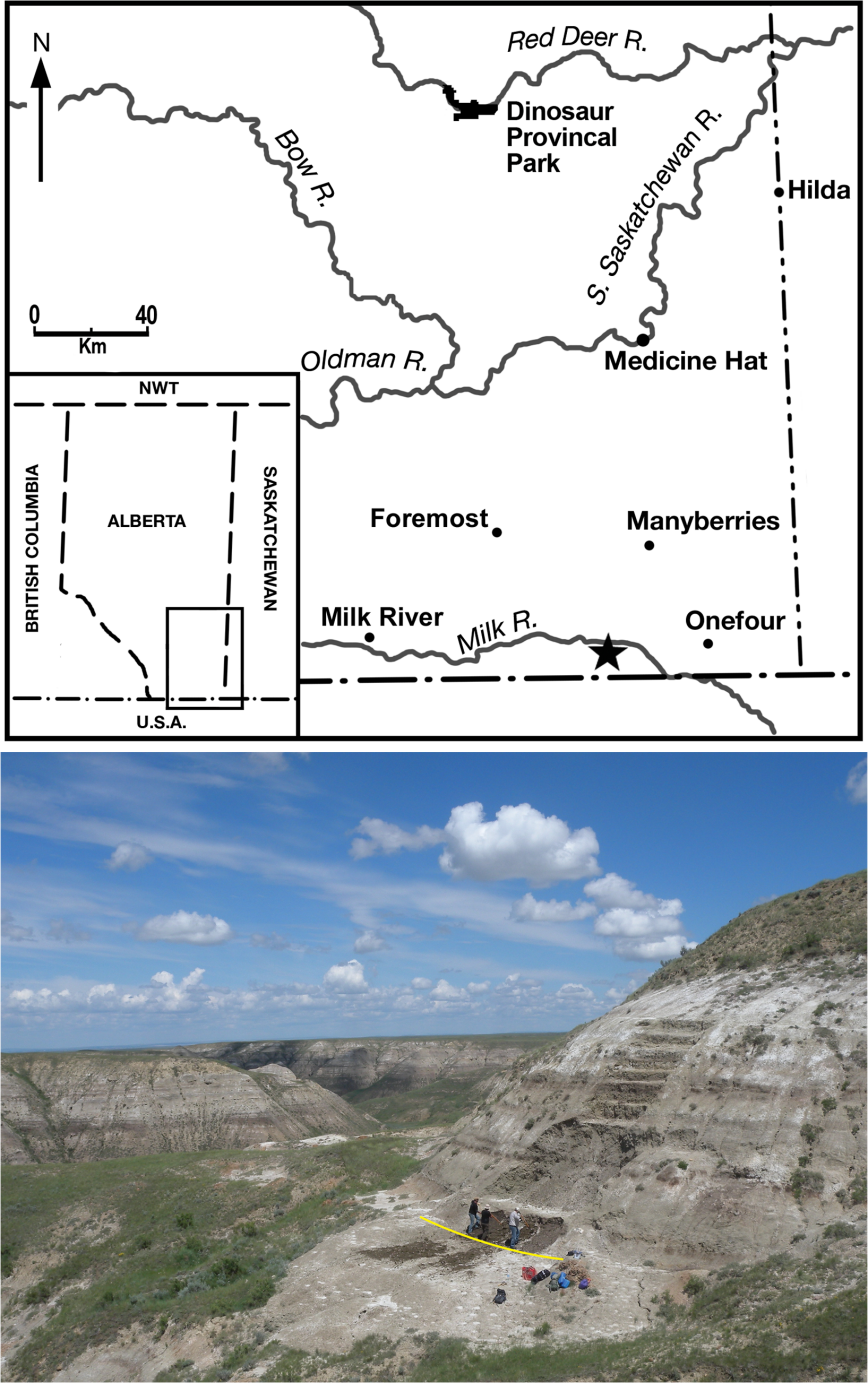
Location and quarry for *Wendiceratops pinhornensis* gen. et sp. nov. Locality map (top) of southern Alberta, Canada, showing the location of the bonebed (indicated with a star) and a photograph of the quarry (bottom); yellow line indicates approximate level of the bonebed at the base of the hill. The west side of the bonebed is its erosional face.

**Fig 2:**
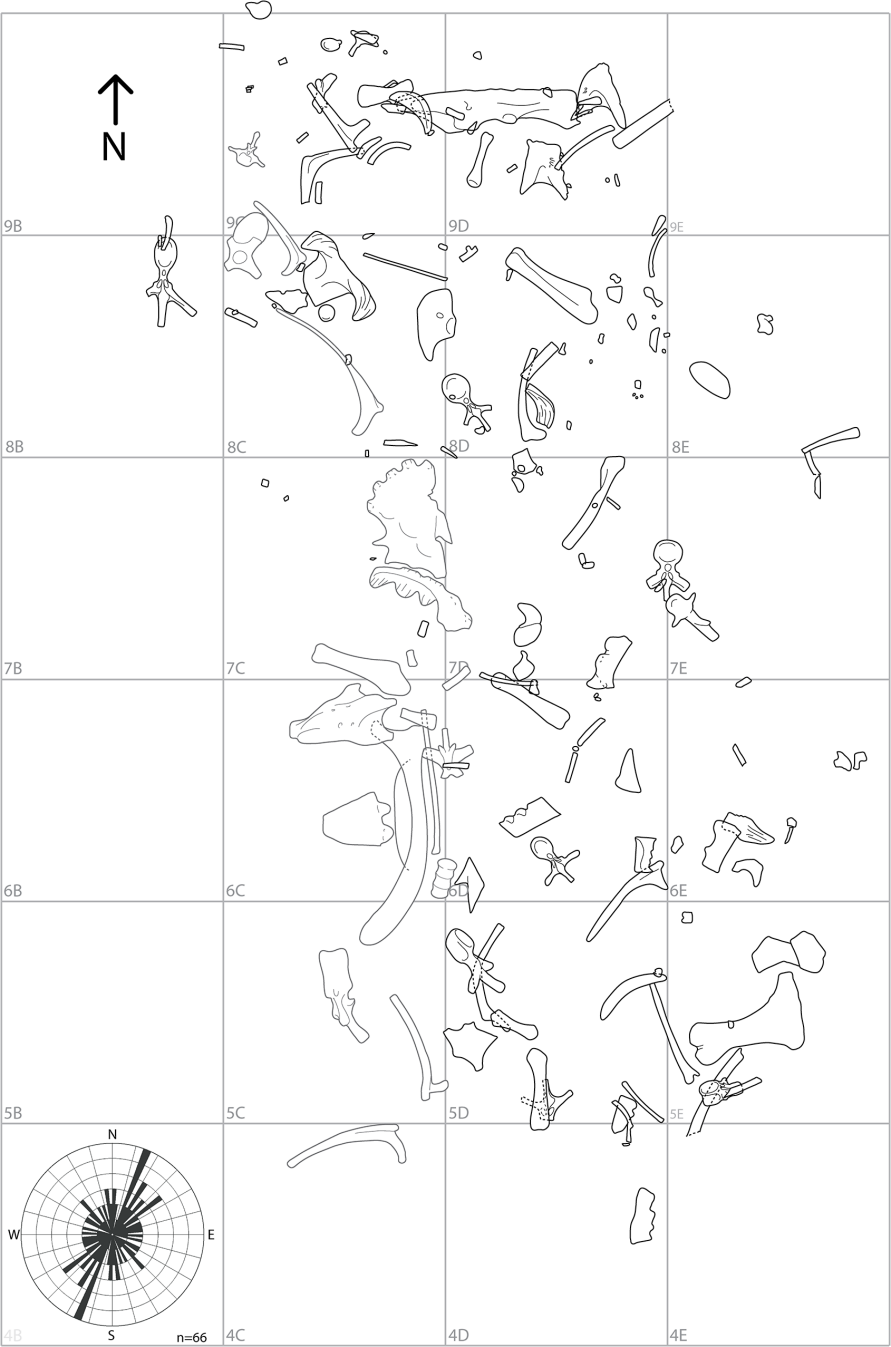
Quarry map for the South Side Ceratopsian bonebed. This bonebed has produced all of the known *Wendiceratops pinhornensis* gen. et sp. nov. material to date. Elements outlined in gray were collected in 2011 and their positions are approximate relative to the more precisely mapped bones collected in 2013 and 2014 (outlined in black). Inset rose diagram denotes a slight NE-SW trend in long axis orientation for the elements over 100 mm in length. See text for comments on the taphonomy of the bonebed.

**Fig 3:**
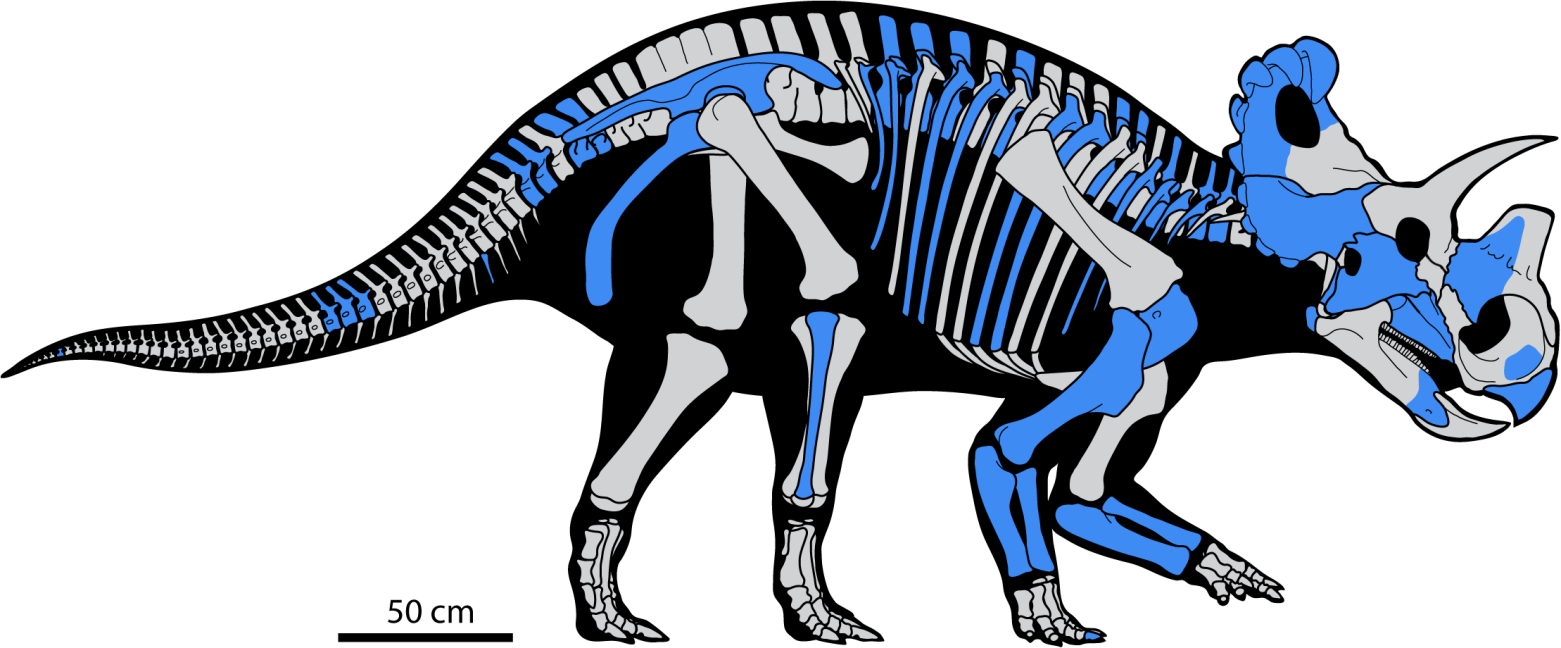
Skeletal reconstruction of *Wendiceratops pinhornensis* gen. et sp. nov. Elements represented in the material collected from the bonebed are indicated in blue.

Here we report on a new species of highly adorned centrosaurine ceratopsid, *Wendiceratops pinhornensis* gen. et sp. nov., from the lower part of the Oldman Formation, Alberta, Canada. Fossil material of the new taxon was collected from a medium-density monodominant bonebed that dates to the middle of the Campanian (approximately 79 Ma), making *Wendiceratops* one of the oldest named members of Ceratopsidae (Figs 1 and 2). The material includes over 200 different bones derived from virtually all parts of the skeleton, including multiple, well-preserved specimens that contribute to the ornamented parietosquamosal frill (Fig 3). The abundance of material makes the new taxon one of the best-represented early ceratopsids. Here we describe the cranial material of the new taxon, which provides additional information on the early diversification of centrosaurines and evolution of cranial ornamentation that characterizes the group, including the earliest evidence of a prominent nasal horn in Ceratopsidae.

### Geologic and Taphonomic setting

The bonebed is located within the boundaries of the Pinhorn Provincial Grazing Reserve, south of the Milk River, Alberta, Canada (Fig 1), and is informally called the “South Side Ceratopsian” bonebed by field crews in reference to its geographic location relative to the river. The bonebed occurs in the Oldman Formation, 10 m above the top of the Taber coal zone, and just above a sandy interval of the Foremost Formation referred to as the Herronton sandstone zone [16]. The quarry is in close geographic proximity to the type locality of *Albertaceratops nesmoi*, and is in correlative strata [6]. The top of the combined Taber Coal Zone and the Herronton sandstone zone marks the contact between the terrestrial Oldman Formation and the underlying Foremost Formation, which represents a transitional marginal marine-terrestrial unit deposited during the regressive phase of the Western Interior Seaway [16]. The Oldman Formation can be divided into three informal subunits (e.g., [5]), the lower and upper mudstone-dominated units separated by the middle sandstone dominated unit, which is referred to as the Comrey sandstone zone. The bonebed occurs in the lower half of the lower mudstone-dominated unit, and is lithostratigraphically correlative to the exposed part of the Judith River Formation at Kennedy Coulee in neighboring Montana, approximately 25 km to the southeast [6]. Radiometric dates from the Kennedy Coulee section, which are presumed to be chronostratigraphically correlative to the bonebed section [14], suggest that the *Wendiceratops* bonebed is between 78.7 and 79.0 million years old [17, 18].

The bonebed host unit sits at the top of a 10 m thick succession of interbedded 20–50 cm thick mudstones, and thin siltstones and sandstones (D. A. Eberth, pers. comm. 2015). The fossils are produced from an approximately 40 cm thick, organic-fragment-rich clayey, sandy mudstone that is rich in coalified root traces, both horizontal and vertical, suggesting a saturated or water-logged anoxic deposit. This interpretation is supported by numerous bones exhibiting wet rot and green fractures. The bonebed zone is massive with no apparent sedimentary structures or lamination, indicating a mass sediment flow origin or extensive trampling, or both; thus, the elements may be largely in situ, or minimally reworked (D. A. Eberth, pers. comm. 2015). The latter interpretation is supported by several bones that were recovered in two pieces and subsequently found to reconnect after preparation (e.g., TMP 2011.051.0009 [parietal]; TMP 2011.051.0010 [squamosal]; TMP 2014.029.0100 [humerus]), as well as numerous plunging elements (e.g., TMP 2014.029.0093 [cervical rib] and TMP 2014.029.0087 [a parietal fragment], both which plunge 90 degrees), which suggest trampling. However, most of the fossil bones in the bonebed are oriented parallel with the beds. The rose diagram (Fig 2) shows a slight east of north preferential long-axis orientation for the bones suggesting at least some reworking.

Six 1 m x 1 m grids were completely excavated, while another 12 grid sections were partially excavated, including at least 6 incomplete grids along the erosional face of the quarry (Fig 2). The average number of elements/grid from the completely excavated grids is 17, with a range of 12 (grid 5D) to 23 (grid 8D) (Fig 2). The average length of the mapped elements (excluding bone fragments) is 173 mm (n = 126).

To date, 221 vertebrate elements have been collected from the main quarry area (Fig 2; S1 File), with 95% (n = 210) of these pertaining to Dinosauria. Virtually all of the dinosaur bones (184 of 186) that can be identified to family-level pertain to Ceratopsidae, and represent almost every part of the body (Fig 3). Of these, all of the bones (n = 27) identifiable to lower taxonomic levels pertain to Centrosaurinae, and nine parietal specimens can be attributed to the new taxon based on diagnostic features of the epiparietal ornamentation. We therefore assign all of the ceratopsid elements in the deposit to *Wendiceratops pinhornensis* due to their close association within a single monodominant deposit (sensu [19]), as is typically done in ceratopsid bonebed studies. The bonebed has a MNI of four, based on three adult-sized left maxillae, and the presence of two very small, juvenile tibiae from different sides of the body.

The only other dinosaurs represented in the deposit are theropods (n = 3), with two shed teeth of tyrannosaurids (TMP 2011.051.0043, TMP 2013.020.0003) identifiable to the family level. Non-dinosaurian freshwater vertebrates are present in low abundance, including turtles (n = 3; TMP 2013.020.0004, TMP 2013.020.0057, TMP 2014.029.0063), crocodilians (n = 1, TMP 2013.020.0043), and garfish (n = 6). The bonebed lithosome is also rich in bivalve and large gastropod shell fragments (e.g., TMP 2014.029.0079), as well as carbonized plant fragments and small pieces of amber (e.g, TMP 2014.029.0028, TMP 2014.029.0064).

## Materials and Methods

All of the bones were collected during systematic excavations of the bonebed carried out between 2011 and 2014. Exploratory excavation of the bonebed was carried out in the summer of 2011 and bones were less precisely mapped in this year than those done in 2013 and 2014. The 2012 field season was largely dedicated to overburden removal (Fig 2). Detailed taphonomic data was collected during the 2013 and 2104 field seasons. All of the collected specimens are catalogued in the collections of the Royal Tyrrell Museum of Palaeontology (TMP, Drumheller, Alberta, Canada) and were prepared at the Royal Ontario Museum (ROM, Toronto, Ontario, Canada) between 2012 and 2015.

To assess the systematic position of *Wendiceratops pinhornensis* gen. et sp. nov., the holotype and associated material were combined into a single OTU (operational taxonomic unit) and coded into an expanded and modified version of the data matrix used in the recent analysis of Sampson et al. [11]. The original Sampson et al. [11] data matrix consisted of 20 taxa and 97 characters, to which we added *W*. *pinhornensis* and four new characters (see S2 File, Characters 98–101). In addition, we modified Characters 59 and 61, which describe the epiparietal (ep) morphology at loci ep 2 and ep 3, to include a state that differentiates between rugose, tongue-shaped horncores and greatly elongate, spike-like horncores that, for example, characterize the ep 3 locus in pachyrhinosaurins and some other taxa (e.g., *Styracosaurus*). We also modified Character 25 from a binary to a multistate character by adding the primitive condition of lacking a distinct postorbital horncore. Preliminary homology assessments of the epiossifications that occupy the periphery of the parietosquamosal frill follow the original analysis [11], which is discussed in Loewen et al. [20] and Clayton et al. [21]. The final data matrix consists of 101 characters and 25 taxa, with 17 in-group centrosaurine taxa (see S3 File).

The cladistic analysis was performed using the software program TNT v. 1.0 [22]. *Leptoceratops gracilis* was designated the outgroup, and characters were run equally weighted, except for Character 20, which was considered ordered following the original analysis. The analysis included a 1000 replicate random addition traditional search with TBR branch swapping holding 10 trees at each replicate, followed by an additional round of TBR branch swapping. In order to assess the robusticity of the topological results, a Bootstrap analysis (1000 replicates) was conducted and Bremer Decay values were calculated using these options in TNT.

### Permits

The material described in this paper was collected following the Historical Resources Act of Alberta (Canada) under Government of Alberta Permit To Excavate Palaeontological Resources Permit Nos. 3950-E03, 12–008, 13–013, 14–033 (DCE) and Permit Nos. 12–034, 13–045, 14–036 (MJR). All necessary permits were obtained for the fossil material described in this study, which complied with all relevant regulations as well as the PLoS Paleontological Ethics Statement. All of the material described in this study is reposited at the Royal Tyrrell Museum of Palaeontology (TMP), Drumheller, Alberta, Canada.

### Nomenclatural Acts

The electronic edition of this article conforms to the requirements of the amended International Code of Zoological Nomenclature, and hence the new names contained herein are available under that Code from the electronic edition of this article. This published work and the nomenclatural acts it contains have been registered in ZooBank, the online registration system for the ICZN. The ZooBank LSIDs (Life Science Identifiers) can be resolved and the associated information viewed through any standard web browser by appending the LSID to the prefix "http://zoobank.org/". The LSID for this publication is: urn:lsid:zoobank.org:pub:789B7DDD-5074-4DF9-A654-D55A4874012C. The electronic edition of this work was published in a journal with an ISSN, and has been archived and is available from the following digital repositories: LOCKSS (http://www.lockss.org); PubMed Central (http://www.ncbi.nlm.nih.gov/pmc).

## Results

### Systematic Palaeontology

Dinosauria Owen, 1842 [23]

Ornithischia Seeley, 1887 [24]

Ceratopsia Marsh, 1890 [25]

Neoceratopsia Sereno, 1986 [26]

Ceratopsidae Marsh, 1888 [27]

Centrosaurinae Lambe, 1915 [28]

*Wendiceratops* gen. nov.

urn:lsid:zoobank.org:act:BEE8D424-8BB0-4335-BFFC-701E2C8B4DB3

#### Diagnosis

Monotypic, as for species.

*Wendiceratops pinhornensis*, gen. et. sp. nov.

urn:lsid:zoobank.org:act:22F3BE83-6045-489D-8F2D-FE53FC08A175

#### Etymology

The generic name honors Wendy Sloboda, who discovered the type locality, combined with *ceratops* (horned-face) from the Greek, a common suffix for horned dinosaur generic names. The specific epithet refers to the Pinhorn Provincial Grazing Reserve in Alberta, Canada, where the type locality is located.

#### Holotype

**Fig 4:**
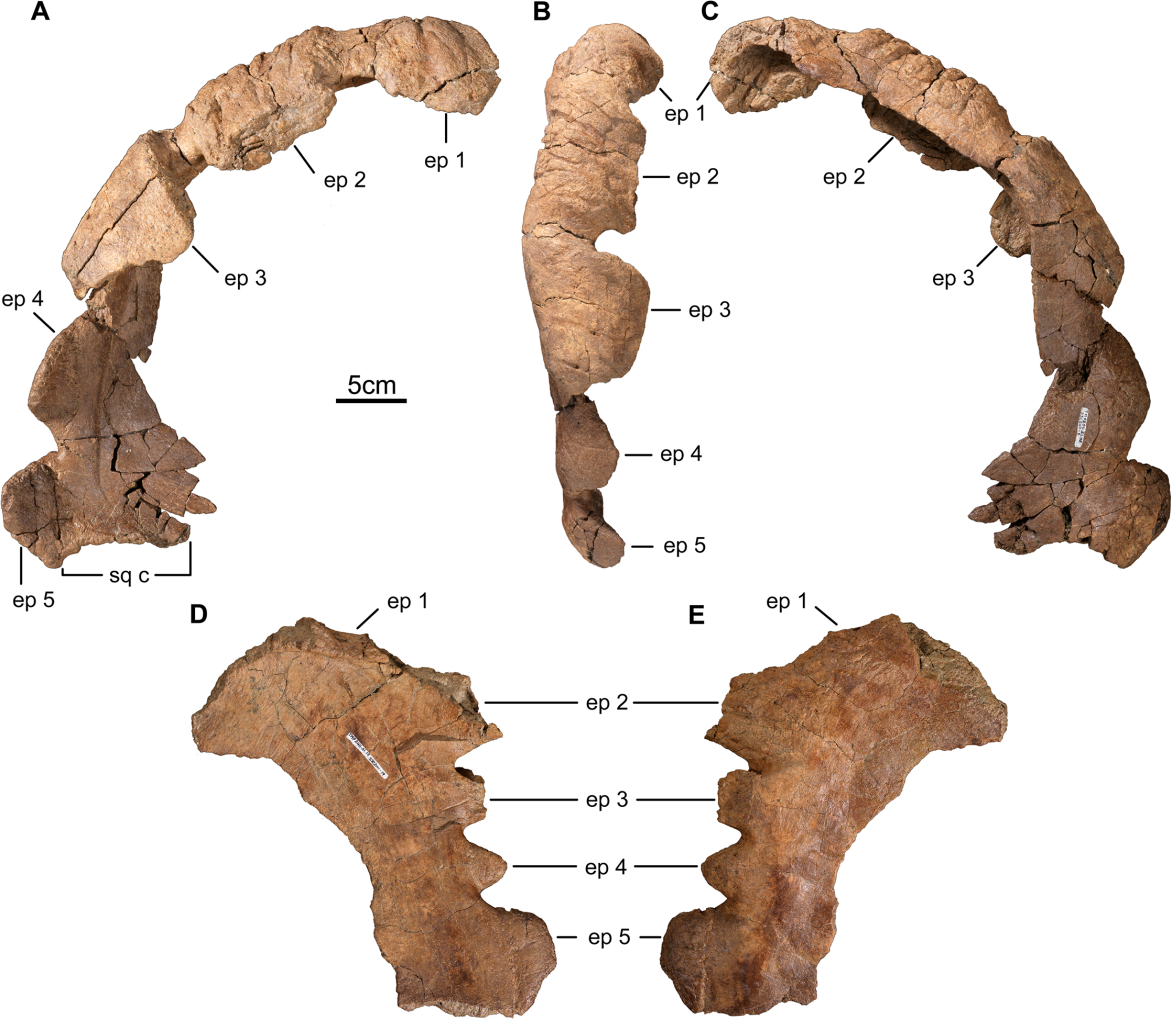
Parietal of *Wendiceratops pinhornensis* gen. et sp. nov. TMP2011.051.0009 (holotype) right parietal ramus in A) dorsal, B) lateral, and C) ventral views. TMP2011.051.0019, subadult left parietal ramus, in D) dorsal and E) ventral views. Abbreviations: ep, epiparietal; sq c, squamosal contact.

TMP 2011.051.0009, an incomplete parietal lacking the midline bar and left ramus (Fig 4A–4C).

#### Referred Material

**Fig 5:**
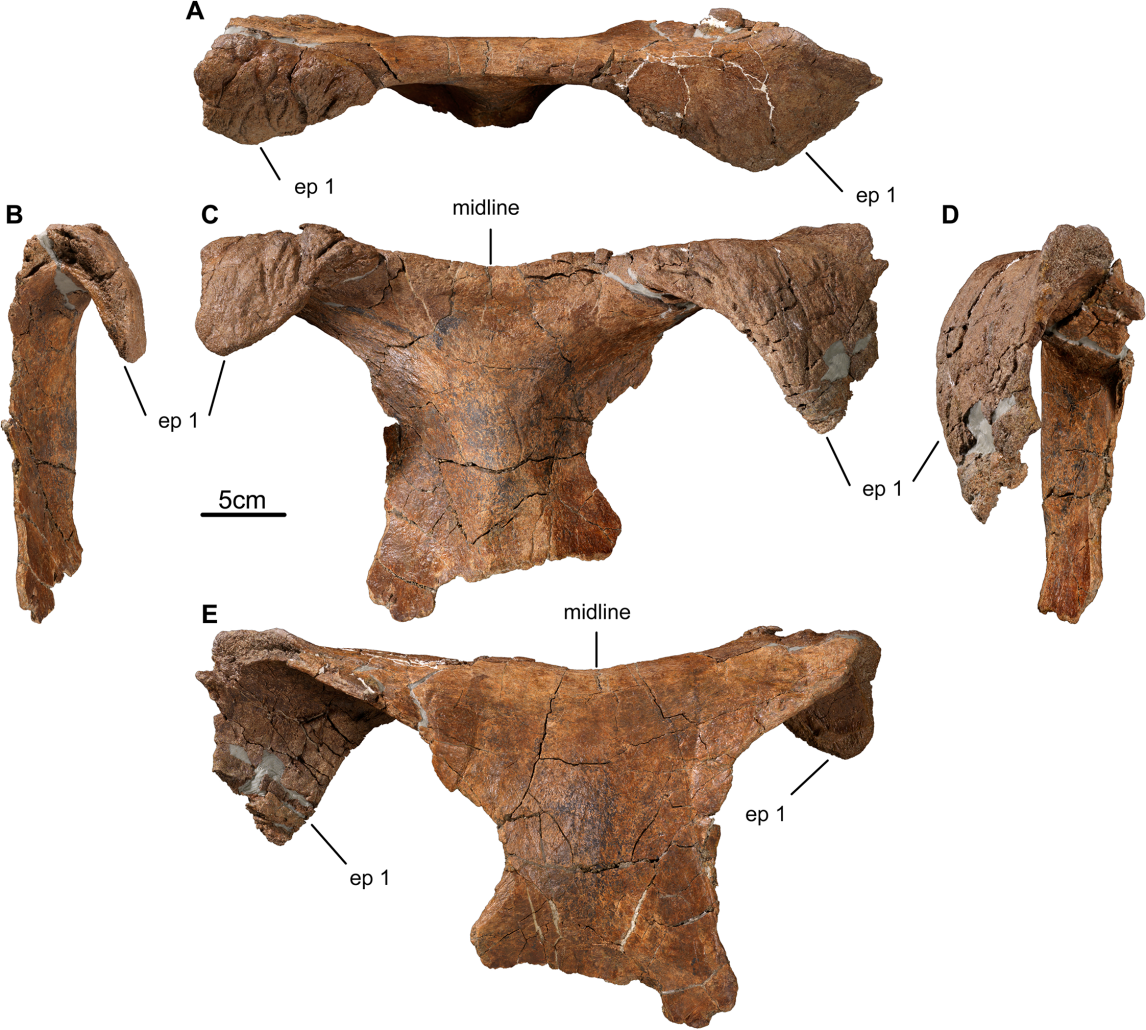
Parietal midline ramus of *Wendiceratops pinhornensis* gen. et sp. nov. TMP 2013.020.0048 in A) posterior (ventral side towards top of page), B) left lateral, C) dorsal, D) right lateral, and E) ventral views. Abbreviation: ep, epiparietal.

**Fig 6:**
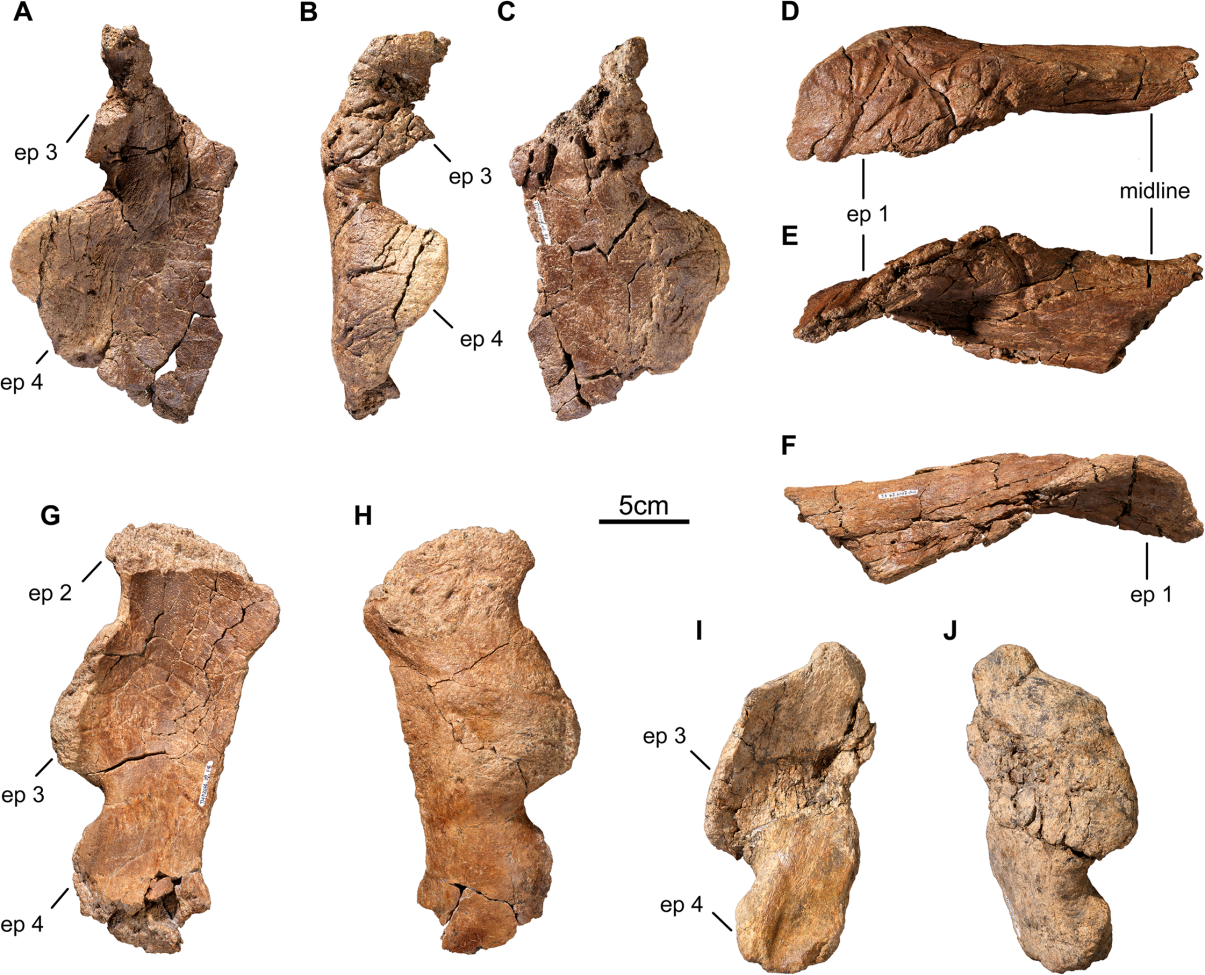
Parietal fragments of *Wendiceratops pinhornensis* gen. et sp. nov. TMP 2014.029.0097 in A) dorsal, B) lateral, and C) ventral views. TMP 2014.029.0015 in D), posterior (ventral side towards top of page), E) dorsal and F) ventral views. TMP 2014.029.0094, parietal fragment, in G) dorsal, and H) ventral views. TMP 2014.029.0016, parietal fragment, in I) dorsal, and J) ventral views. Basal width of the laterally positioned epiparietals appears to be variable between individuals, as is the degree of dorsal curvature of ep 4. Abbreviation: ep, epiparietal.

**Fig 7:**
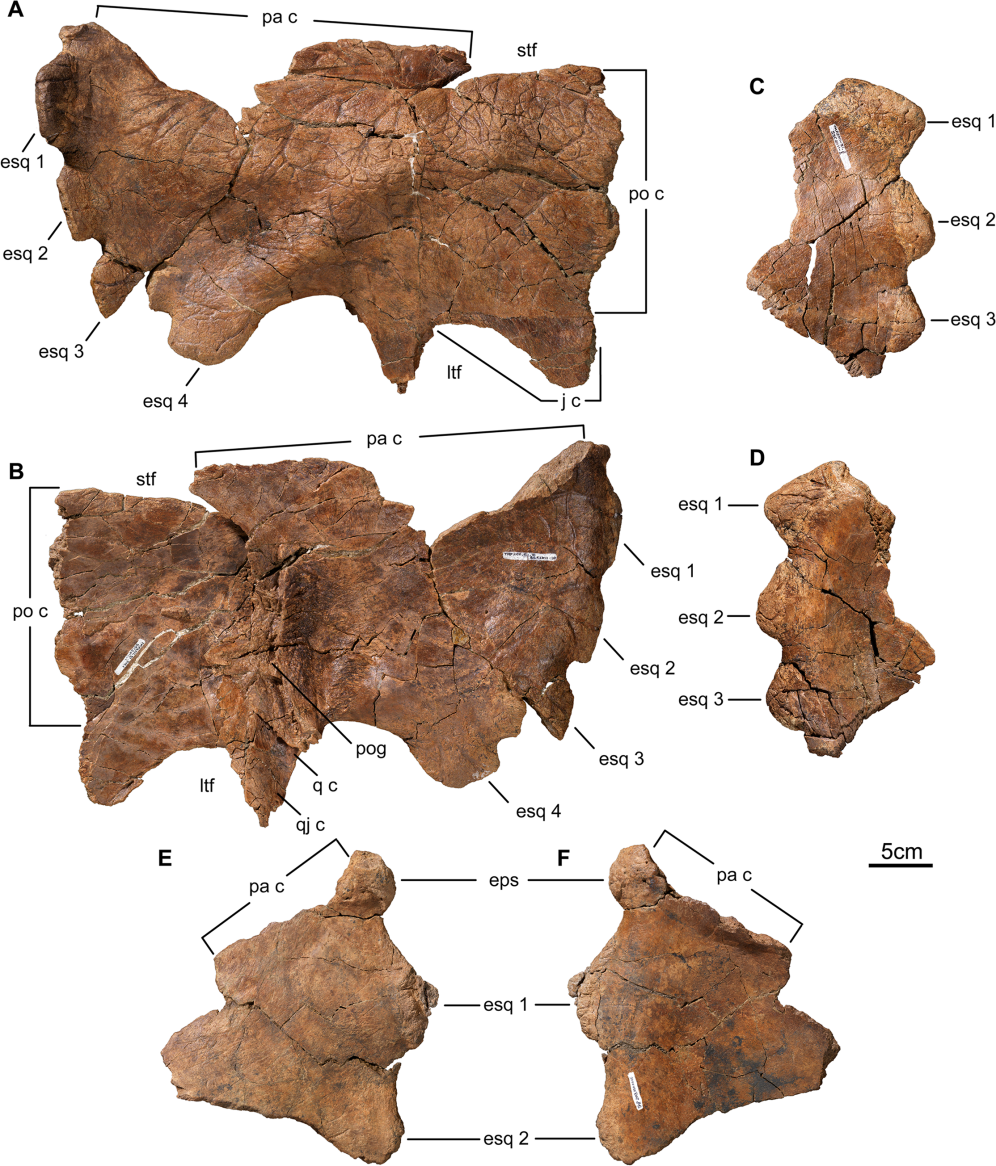
Squamosal of *Wendiceratops pinhornensis* gen. et sp. nov. TMP 2011.051.0001, right squamosal, in A) dorsal and B) ventral views. TMP 2011.051.0002, squamosal fragment, in C) dorsal and D) ventral views. TMP 2013.020.0006, squamosal fragment, in E) dorsal and F) ventral views. Abbreviations: esq, episquamosal; ltf, lateral temporal fenestra; j c, jugal contact; pa c, parietal contact; po c, postorbital contact; pog, paroccipital groove; q c, quadrate contact; qj c, quadratojugal contact; stf, supratemporal fenestra.

**Fig 8:**
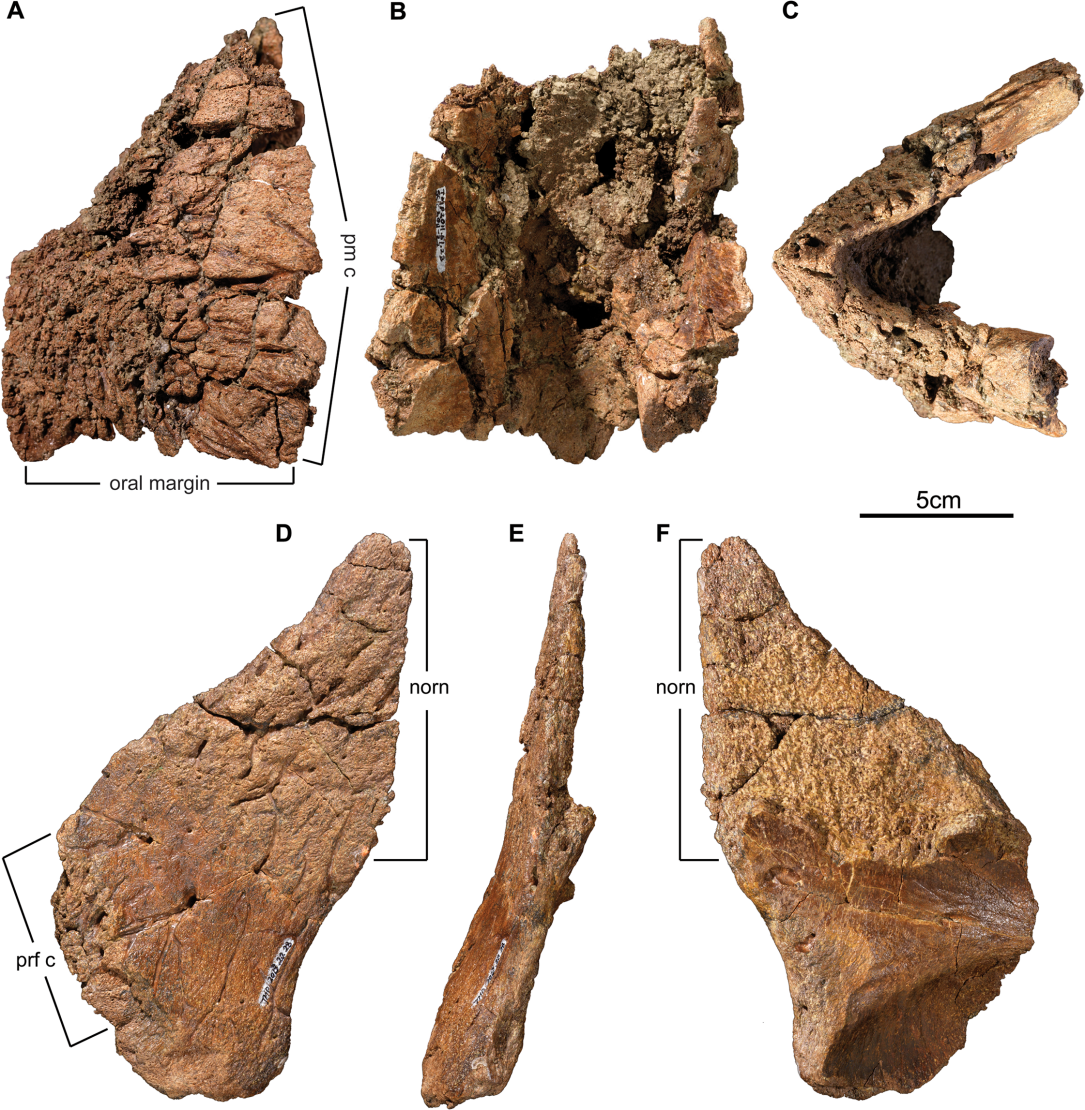
Rostral and nasal of *Wendiceratops pinhornensis* gen. et sp. nov. TMP 2011.051.0023, fragmentary rostral, in A) left lateral, B) posterior, and C) ventral views. TMP 2013.020.0028, right nasal fragment, in D) right lateral, E) anterior, and F) internal views. The preserved nasal ornamentation (norn) represents only the lower portion of what is inferred to be a prominent, erect horncore. Abbreviations: norn, nasal ornamentation; pm c, premaxilla contact; prf c, prefrontal contact.

**Fig 9:**
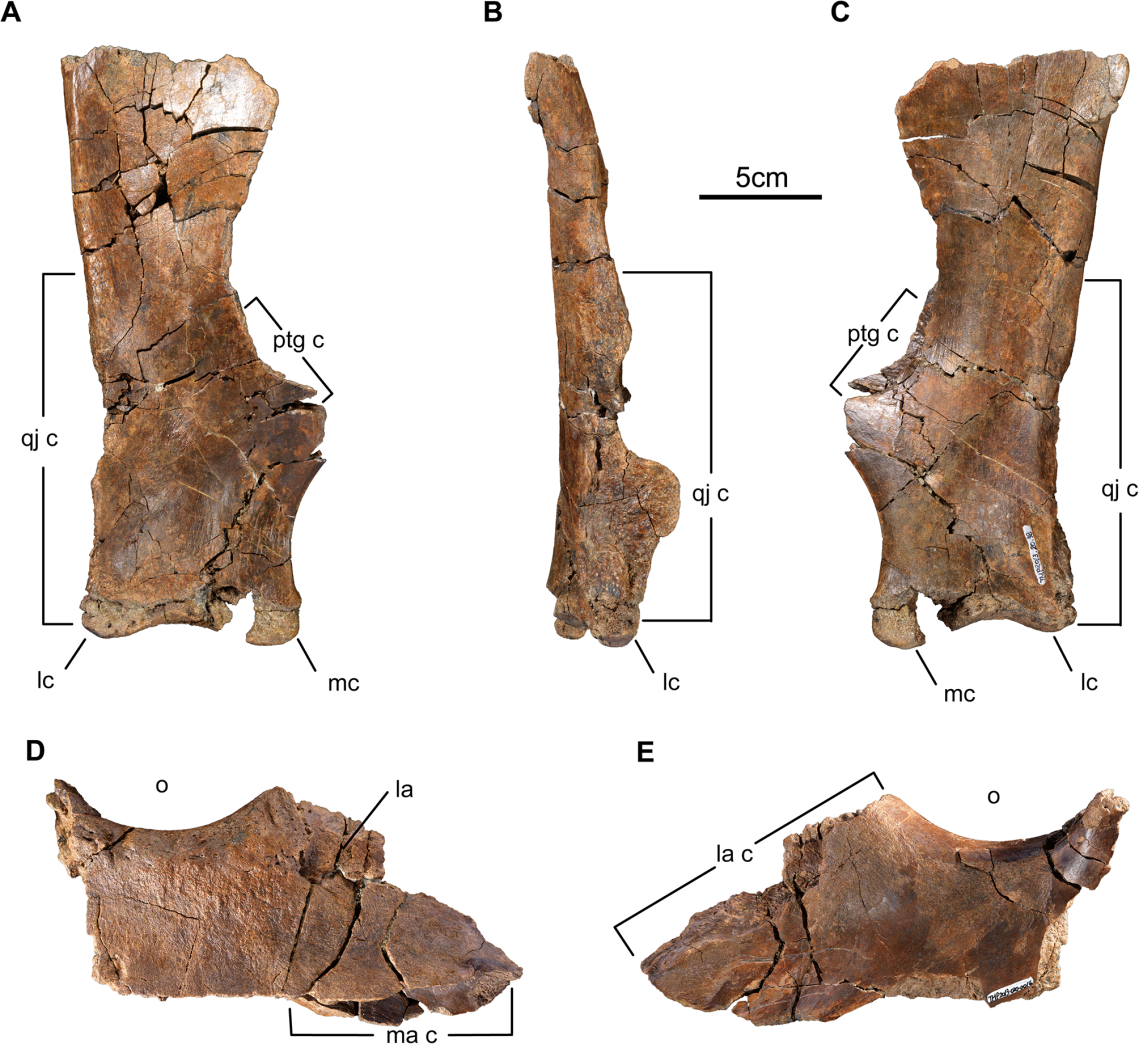
Quadrate and jugal of *Wendiceratops pinhornensis* gen. et sp. nov. TMP 2013.020.0058, right quadrate, in A) anterior, B) lateral and C) posterior views. TMP 2013.020.0016, right jugal, in D) dorsal and E) ventral views. Abbreviations: mc, medial condyle; la, lacrimal; lc, lateral condyle; ma c, maxilla contact; o, orbit; qj c, quadratojugal contact; ptg c, pterygoid contact.

**Fig 10:**
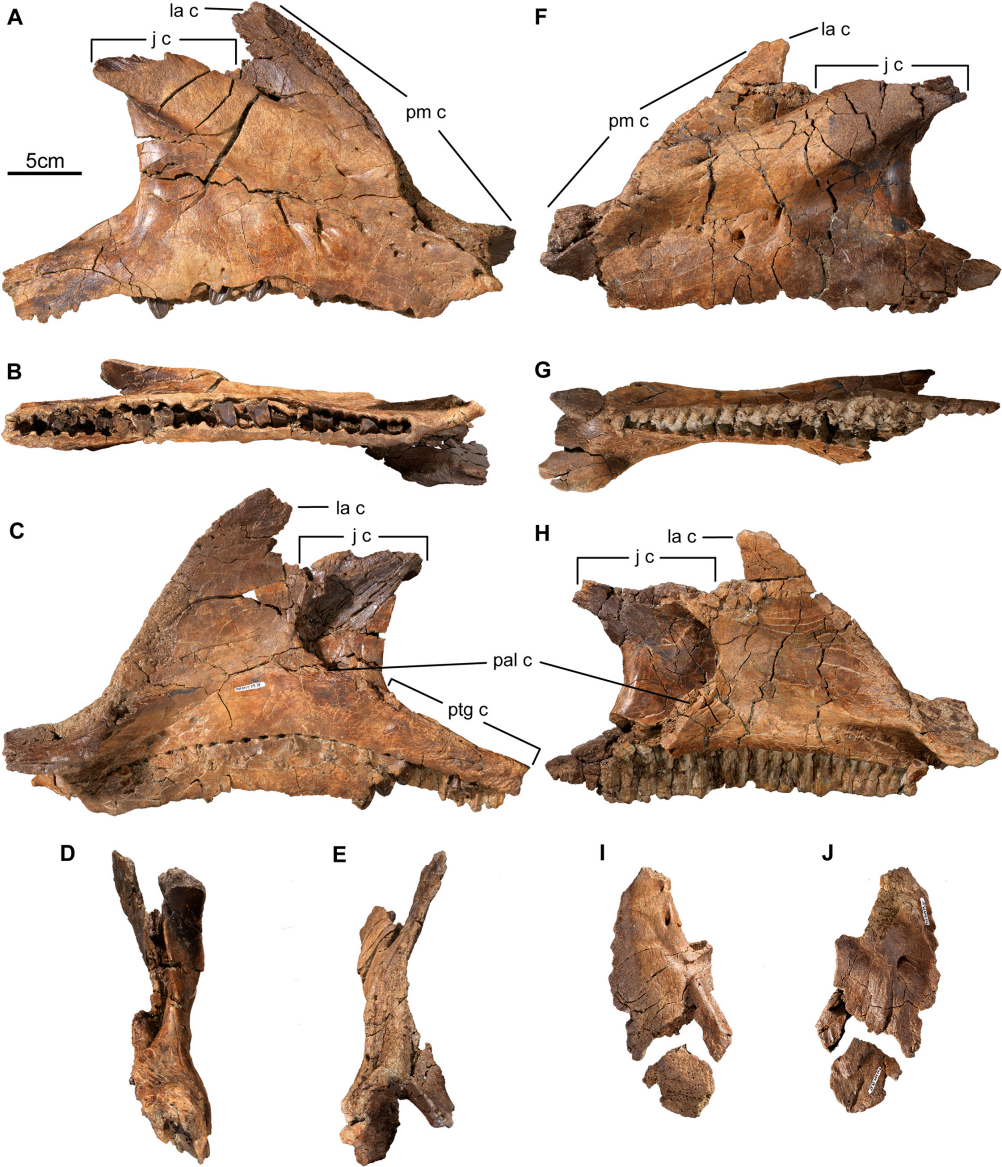
Premaxilla and maxilla of *Wendiceratops pinhornensis* gen. et sp. nov. TMP 2014.029.0074, right maxilla, in A) lateral, B) ventral, C) medial, D) posterior, and E) anterior views. TMP 2011.051.0036, left maxilla, in F) lateral, G) ventral, and H) medial views. The maxilla lacks an accessory antorbital fenestra and the toothrow is ventrally offset relative to the anterior edentulous process. TMP 2014.029.0067, premaxilla fragment, in I) lateral, and J) medial views. Abbreviations: j c, jugal contact; la c, lacrimal contact; pal c, palatine contact; pm c, premaxilla contact; ptg c, pterygoid contact.

Cranial material referred to *Wendiceratops pinhornensis* gen. et sp. nov. includes: TMP 2011.051.0019 (subadult lateral parietal bar; Fig 4D and 4E); TMP 2013.020.0048 (parietal, posterior midline bar; Fig 5); TMP 2014.029.0097 (parietal fragment; Fig 6A–6C); TMP 2014.029.0016 (parietal fragment; Fig 6I and 6J), TMP 2011.051.0010 (right squamosal; Fig 7A and 7B), TMP 2011.051.0002 (squamosal fragment; Fig 7C and 7D); TMP 2013.020.0006 (squamosal fragment; Fig 7E and 7F); TMP 2013.020.0035 (incomplete pathological squamosal); TMP 2013.020.0028 (nasal fragment; Fig 8D–8F); TMP 2013.020.0016 (incomplete jugal; Fig 9D and 9E); TMP 2014.029.0074 (right maxilla, Fig 10A–10E). For a complete list of referred material, see Supporting Information (S1 File).

#### Locality, horizon and age

The type locality occurs within the boundaries of the Pinhorn Provincial Grazing Reserve, south of the Milk River, County of Forty Mile No.8, Alberta, Canada (Fig 1). All of the material was collected from a medium-density monodominant ceratopsid bone bed hosted within an approximately 40 cm thick gray-brown, carbonaceous mudstone layer. The bonebed horizon occurs 10 m above the top of the Taber Coal Zone and within the lower mud-dominated unit of the Oldman Formation (sensu [5]). This unit is lithostratigraphically correlative to the Judith River Formation exposed in Kennedy Coulee, Montana [6], with the top of the Marker A Coal in the Judith River Formation being equivalent to the top of the Taber Coal Zone in the Belly River Group of Alberta. Given the close geographic proximity of these two areas we presume they are also chronostratigraphic correlates, as in previous studies [14]. Therefore, the two radiometric dates of 79.0 Ma and 78.7 Ma from bentonites that occur 5 m below and 27 m above the top of the Marker A Coal, respectively, in Kennedy Coulee [17, 18] constrain the age of the *Wendiceratops* bonebed to within this time interval. Precise locality data is on file with the Royal Tyrrell Museum of Palaeontology.

#### Diagnosis

Centrosaurine ceratopsid that exhibits the following autapomorphies: epiparietals at loci ep 2 and ep 3 developed as broad-based, pachyostotic processes that are procurved anterodorsally to overhang the posterior and lateral parietal rami; ischium with a broad, rectangular distal terminus. Differs from *Diabloceratops*, *Nasutuceratops*, and *Albertaceratops*, in having an upright nasal horncore, although its precise morphology remains unknown. Differs from *Styracosaurus*, *Rubeosaurus* and pachyrhinosaurins in lacking an elongate spike-like process at any parietal locus. *Wendiceratops* shares with *Sinoceratops* the general arrangement of epiparietal processes at ep 1–4 loci that curve anterodorsally, but lacks a posterior midline epiparietal (ep 0) and dorsal eminences on the posterior parietal ramus below the epiparietals.

#### Comments

The bonebed was discovered by Wendy Sloboda in 2010, and excavated during the summers of 2011 through 2014. The holotype lateral parietal ramus consists of two pieces that were found separately, but closely associated in the deposit. TMP 2011.051.0009, the larger section that has well-developed and diagnostic ornamentation, was found to re-connect with a smaller parietal fragment (TMP 2011.051.0005) that includes the contact for the squamosal after preparation. The recombined pieces are referred to here as TMP 2011.051.0009. The holotype parietal, the posterior parietal midline bar (TMP 2013.020.0048), and the almost complete squamosal (TMP 2011.051.0001 with TMP 2011.051.0010) were all found closely associated (within approximately 2 square meters) (Fig 2). The remainder of the material is assigned to the new taxon due to close association with the type specimen within the same monodominant centrosaurine ceratopsid bonebed.

### Description

In total, 12 different skull bones are represented in the sample. Most are fragmentary, such as the premaxilla and dentary, and not diagnostic below Ceratopsidae. Unfortunately, most of the braincase (except a juvenile exoccipital) and the postorbital have not been recovered. However, the recovered material includes several parietals and squamosals that show species-level diagnostic characters. We describe the skull elements in detail below, but provide only a brief overview of the appendicular elements recovered from the bonebed. A detailed description of the postcranial bones will be presented in a separate publication. We first describe the bones that compose the parietosquamosal frill, starting with the holotype parietal, followed by the rest of the cranium and lower jaws.

#### Parietal

The holotype parietal (TMP 2011.051.0009; Fig 4A–4C) consists of the incomplete posterior bar and the largely complete right ramus. The posterior ramus is broken along the first preserved epiparietal (ep), and the posterior margin forms a semicircular outline in dorsal view. The surface texture of the bone is similar to that noted for adult centrosaurines [29], suggesting the specimen was derived from a mature individual with fully developed parietal ornamentation. Laterally, the proximolateral surface terminates as a transversely-oriented surface for contact with the squamosal. The margin adjacent to the parietal fenestra is broken and incomplete throughout its length. Five fused epiparietal processes are preserved on the posterior margin of TMP 2011.051.0009. Only the lateral portion of medialmost epiparietal is preserved. Based on the size of the specimen, the morphology of the midline bar (TMP 2013.020.0048), and comparisons to other taxa, we hypothesize that this represents the first epiparietal locus lateral to the midline (ep 1). The medial three epiparietals (ep 1–3) are pachyostotic and strongly procurved to form broad, rugose horns that project forward and overhang the parietal fenestra. The basal widths of epiparietals 3–5 decrease laterally (ep 2: 101 mm; ep 3: 102 mm; ep 4: 94 mm; ep 5: 82 mm), with the lateralmost epiparietal process adjacent to the dorsoventrally thickened contact region for the squamosal being the smallest process in the series and only incipiently curved anterodorsally. The apices of ep 1–3 are not complete distally, and therefore would have been longer prior to breakage. The dorsal surface of the parietal below the epiparietals is distinctly concave anteroposteriorly. This contrasts with the same region of the parietal in *Sinoceratops*, in which a series of dorsal eminences occur proximal to the base of the epiparietals [30]. Although numerous centrosaurine taxa have strongly procurved pachyostotic ep 1 processes (e.g., *Centrosaurus*, *Coronosaurus*, *Xenoceratops*), the prominently developed and strongly procurved ep 2 and ep 3 processes are unique within Centrosaurinae. The broad concavity of the lateral parietal bar below the first three epiparietals is also different from all other ceratopsids, but is likely related to the extreme forward curvature of the associated epiparietal ornamentation, since the depressed region is absent on immature specimens that lack strongly procurved epiparietals (e.g., TMP 2011.051.0019). The tapering morphology of the medial region of lateral and posterior parietal bar suggests the presence of a large parietal fenestra.

TMP 2011.051.0019 (Fig 4D and 4E) is an incomplete left parietal ramus that preserves the posterolateral margin with parts of five fused epiparietals. The specimen is relatively small, has long-grained bone texture around the margin of the parietal fenestra, and has incipiently developed ornamentation; all suggesting that the specimen represents a subadult individual [29]. The complete epiparietals have variable basal widths (ep 2: 110mm; ep 3: 40 mm; ep 4: 40 mm; ep 5: 79 mm), but have generally the same proximodistal height where completely preserved. Only the lateral part of the base of ep 1 is preserved, but it is clearly a procurved process. The base of ep 2 is complete. It is broken distally, but enough is preserved to show that it was apparently large and weakly procurved. The ep 3 and ep 4 processes have short, thickened bases, but are not developed into the exaggerated, strongly procurved, tongue-shaped processes that characterize the larger, more mature specimens. The differences in the morphology and relative prominence of the epiparietal processes between the subadult TMP 2011.051.0019 and the more mature holotype are interpreted as the result of ontogenetic variation in which the relative size of the processes are positively allometric. This pattern of epiparietal growth occurs frequently in centrosaurines [31].

A single posterior midline bar (TMP 2013.020.0048; Fig 5) was collected. The bone surface texture suggests it pertained to an adult [29]. As preserved, it measures 405 mm across its posterior margin, with a midline length of 85 mm. It has a shallow embayment (approximately 170°) between the medial surfaces of the preserved ep 1 processes, which are separated by 150 mm. The right ep 1 process is broken and incomplete laterally and distally, whereas the left ep 1 is almost complete. The base of the left ep 1 process measures 165 mm mediolaterally, and projects anteriorly 98 mm from the transverse posterior ramus. It is roughly triangular in dorsal view, with its main axis directed slightly laterally. The midline bar is transversely broad. There is a weak midline ridge that lacks dorsal scalloping (as in *Centrosaurus apertus*, e.g., ROM 767). The thin, tapering lateral margins on the either side of the midline, as well as morphology of the holotype lateral parietal bar, suggest that the frill had well-developed fenestrae, as in most ceratopsids.

Numerous other broken pieces of parietal preserving the diagnostic ornamentation have been recovered from the bonebed (Fig 6). TMP 2014.029.0094 (Fig 6G and 6H) is a large fragment of lateral parietal bar with parts of three incomplete epiparietals fused to its posterior margin. Based on comparison with the TMP 2011.051.0009, we interpret the epiparietals to represent ep 2–4. The medial region of the dorsal surface of the posterior bar, corresponding to the position of ep 2, exhibits the long-grain surficial bone texture that characterizes immature ceratopsid parietals [29]. The lateralmost epiparietal is incomplete, but it is clearly smaller than the other two epiparietals and appears to be developed as a low, D-shaped process. The more medial epiparietals are broad-based, procurved, pachyostotic processes that are incomplete distally. The central epiparietal, interpreted as ep 3, has a complete base that measures 100 mm in length. As in the holotype, the dorsal surface of the parietal below the epiparietals is smooth and weakly concave anteroposteriorly.

#### Squamosal

The squamosal is represented by multiple fragments (e.g., TMP 2011.051.0014, TMP 2013.020.0006; Fig 7), many of which preserve portions of the peripheral ornamentation, as well as an almost complete right squamosal (TMP 2011.051.0001; Fig 7A and 7B). TMP 2011.051.0001 has the typical rectangular centrosaurine shape (455 mm, preserved length). The anterior contact with the postorbital is complete, but much of the posteromedial contact with the parietal is broken away. The squamosal has the diagnostic 'stepped-up' morphology of centrosaurines [32]. However, *Wendiceratops* differs from the basal centrosaurine *Diabloceratops* in its approximately 1:1 height to length ratio, and in having more than two episquamosals (es). The posterior margin of TMP 2011.051.0001 has four episquamosal loci, all of which are occupied by well-fused episquamosals. Episquamosals 1–3 are dorsally inflected, and, as preserved, have basal lengths approximately twice their heights. The episquamosals on this specimen appear to be squared off distally, but this impression is exaggerated by the erosional loss of their apecies. The episquamosals of other specimens have a slightly asymmetrical triangular shape (e.g., TMP 2011.051.0002, TMP 2013.020.0006, Fig 7). A small epiossification that caps the squamosal-parietal contact (eps) is present in TMP 2013.020.0006 (Fig 7E and 7F). The jugal notch is wide and the jugal process is incomplete ventrally. The anterodorsal margin of the infratemporal fenestra preserves an overlapping contact on the dorsal surface of the squamosal indicating that the jugal formed the anterior margin of this opening.

The dorsal surface of the squamosal is notable in having a well-developed ridge that extends from the anteromedial margin to the base of es 4. This ridge is formed by the coalescence of three prominent, dorsally rugose bumps that can be found to varying degrees on all ceratopsids, but are most prominent on basal centrosaurines where they typically develop a similar ridge-like eminence (e.g., *Nasutuceratops*, *Avaceratops*). Although most of the posteromedial margin that would have contacted the parietal is not preserved, the thickness of the squamosal in this region indicates that it was not expanded as for some centrosaurines (e.g., Pachyrhinosaurini [33]). As for all ceratopsids, the dorsal surface is impressed with numerous vascular grooves, most of which originate near the medial margin, and the ventral surface is relatively smooth.

#### Rostral

The single rostral in the sample (TMP2011.051.0023; Fig 8A–8C) is heavily damaged by modern erosion on the left side. Both the dorsal and subnarial processes are incomplete. Unfortunately, the overall shape of the bone is difficult to discern due to incompleteness, but the rostralmost margins of the bone form a weakly decurved beak. The external surface is densely penetrated by vascular foramina, as is typical of ceratopsids. In contrast, the medial surfaces consist of smooth cortical bone, and the contact surfaces for the premaxilla are present on preserved parts of the posteromedial margin (Fig 8B).

#### Maxilla

Four maxillae, TMP 2011.051.0036, TMP 2013.020.0025, TMP 2014.029.0074, TMP 2014.029.0095, were recovered. TMP 2014.029.0074 (Fig 10A–10E) is the most complete (350 mm, preserved length), and has at least 26 alveoli, with numerous replacement teeth in place. TMP 2011.051.0036, from a slightly smaller individual, is also particularly well preserved, but it lacks dentition (Fig 10F–10H). The maxilla is approximately triangular in shape, with a ventral horizontal ramus that forms the body of the bone and includes the tooth row, and an ascending ramus that contacts the premaxilla rostrally, as well as the jugal and lacrimal dorsally. The anterior region of the bone consists of a transversely expanded anterior shelf that cradles the base of the posterior process of the premaxilla. The contact surface with the premaxilla extends from this point along the thin, posterodorsally oriented anterior margin of the dorsal process. The continuous nature of the premaxillary contact and the flat anterolateral surface of the bone in this area indicate that *Wendiceratops* lacked an accessory antorbital fenestra, as is present in *Diabloceratops eatoni*. The dorsal process bifurcates near its apex to define the margins of a small antorbital fossa, which would have been completed by the lacrimal. Posteriorly, TMP 2014.029.0074 and TMP 2011.051.0036 have a pronounced buccal excavation. The ventral margin of the maxilla that defines the toothrow is ventrally offset relative to the anterior edentulous process that forms the base of the premaxillary shelf. This feature occurs in the basal centrosaurines *Diabloceratops*, *Nasutuceratops*, and *Avaceratops*, but it is absent in more derived centrosaurines such as *Centrosaurus apertus*. Internally, the medial shelf forms the short premaxillary shelf rostrally and narrows to meet the palatine posteriorly. The size of the ectopterygoid on lateral surface of posterior ramus cannot be determined since the contact surface for the ectopterygoid is not completely preserved on any of the known specimens.

The lateral surface of the central body of the maxilla is perforated by numerous, irregularly-sized neurovascular foramina. The medial surface is smooth, and perforated by a concave row of special foramina that correspond to the medial bases of the tooth files that extend deep into the body of the bone. On the posterior half of the bone, a long, rugose scar marks the contacts for the palatine (anteriorly) and pterygoid (posteriorly).

#### Nasal

Parts of three nasals have been collected, but only a single large fragment (TMP 2013.020.0028; Fig 8D–8F) is informative. It consists of a thin cortical portion of the nasal ornamentation dorsally and part of the postnarial apron ventrally. The midline contact with the contralateral nasal is not preserved, thus revealing the spongy, cancellous bone of the broken core of a large nasal horncore. The highly rugose external surface texture is indicative of a subadult or adult individual. The maximum dimension of the fragment is 190 mm, with a preserved length along the base of the horn of 90 mm. The nasal ornamentation is flat externally, and offset from the body of the nasal such that the lateral surface of the horn and the post-narial apron form and angle of approximately 170 degrees in anterior view. (Fig 8E). The height of the nasal ornamentation as preserved is 115 mm, but all surfaces are incomplete, suggesting that the true dimension of the horncore was considerably larger than what is preserved. Below the base of the nasal ornamentation, the lateral surface of the nasal is deeply impressed by two sets of branching vascular valleculae. Homologous vascular impressions occur in other centrosaurines immediately below the posterior base of the nasal horncore (e.g., *Centrosaurus apertus*, ROM 767). The morphology of TMP 2013.020.0028 suggests that the nasal ornamentation consisted of a prominent, upright nasal horncore that was relatively larger than in *Diabloceratops* [9], *Nasutuceratops* [11], and the holotype of *Albertaceratops* [6]. Beyond this, the overall shape of the nasal horn cannot be determined. The posterior margin of the fragment also preserves part of the rugose contact surface for the prefrontal, which it would have met in a thickened interdigitating joint. Based on the proximity of the prefrontal contact to the preserved ornamentation, it appears that the nasal ornamentation in *Wendiceratops* was located close to orbits, as in *Nasutuceratops* [11] and *Albertaceratops* [6]. Medially, a portion of the dorsolateral roof of the nasal cavity is present on the ventromedial surface of the nasal fragment, below the broken spongy bone medial to the horncore.

#### Jugal

Two fragmentary jugals (TMP 2001.051.0012, TMP 2013.020.0016) were recovered, of which TMP 2013.020.0016 is the most complete (Fig 9D and 9E). The incomplete right jugal preserves the ventral margin of the orbit and most of the anterior process, but it is broken posteriorly and is missing the contacts for the postorbital and squamosal. The triangular anterior process contacts the lacrimal dorsally and the maxilla ventrally. A small fragment of the right lacrimal is preserved along the interdigitating contact surface for the lacrimal. The contact for the maxilla is robust and interdigitates posteriorly, and becomes a lap joint where it overlaps the maxilla anteriorly. The thickness of the orbital margin and the sculpturing on its dorsal surface, as well as the overall size of the element, suggests that it was from a large, adult-sized individual.

#### Quadrate

The only preserved quadrate, TMP 2013.20.58 (Fig 9A–9C), from the right side, has a nearly complete body. But as in most isolated ceratopsid quadrates, the majority of the thin pterygoid flange and proximal head that contacts the squamosal is broken away. As preserved, it has a height of 250 mm, and its ventral articular condyle is 88 mm wide. These parameters suggest that the element came from a large, adult-sized animal. The triangular ventrolateral surface of the element is thickened and rugose for contact with the quadratojugal.

#### Lower Jaw

**Fig 11:**
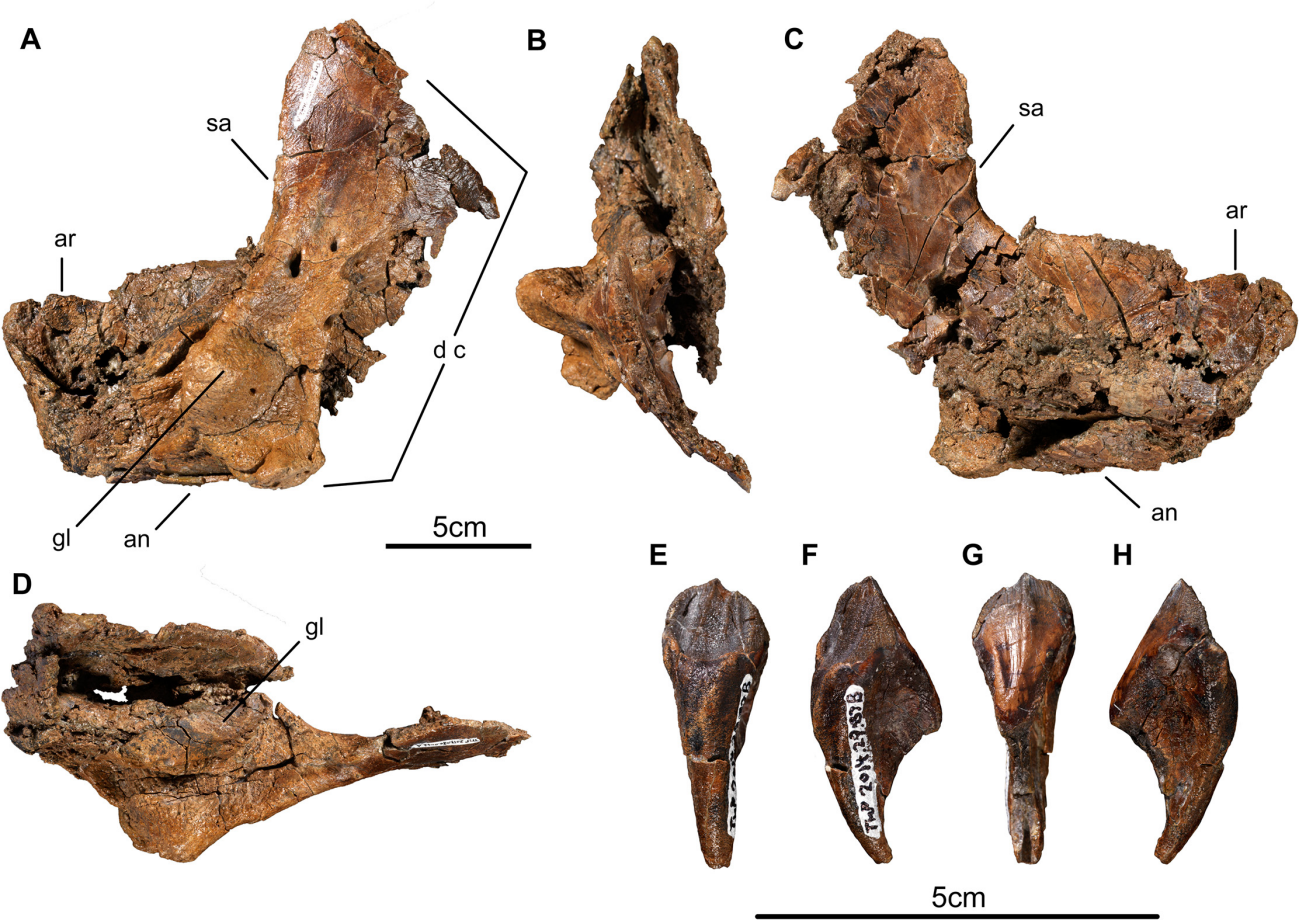
Lower jaw elements and tooth of *Wendiceratops pinhornensis* gen. et sp. nov. TMP 2013.020.0062, left surangular and associated elements, in A) lateral, B) posterior, C) medial and D) dorsal views. TMP 2014.027.0087B, maxillary(?) tooth, in E) labial, F) posterior, G) lingual and H) anterior views. Abbreviations: an, angular; ar, articular; d c, dentary contact; gl, glenoid; sa, surangular.

Only fragments of the dentary have been found. One relatively complete, adult-sized surangular complex, TMP 2013.020.0062 (Fig 11A–11D), was collected from the bonebed. It includes portions of the angular and articular, but it is badly crushed. The preserved material appears to be of typical centrosaurine morphology (e.g., ROM 767, *Centrosaurus apertus*; *Pachyrhinosaurus lakustai*, [33]).

#### Dentition

At least 10 ceratopsian teeth were collected from the bonebed, all of which are typically ceratopsid in their size and morphology. One maxilla, TMP 2014.029.0074, also contains numerous, unworn, replacement teeth. All recovered teeth consist of a leaf-shaped crown with marginal papillae. The enamelled surface has a strong vertical keel that is offset either mesially or distally from the central plane of crown. The keel is flanked by a series of finer subsidiary ridges aligned subparallel to the keel that ornament the enamel surface. One almost complete maxillary tooth, TMP 2014.027.0087B (Fig 11E–11H), preserves both the crown and the root, which has the typical double-rooted ceratopsid morphology.

#### Postcranial Skeleton

**Fig 12:**
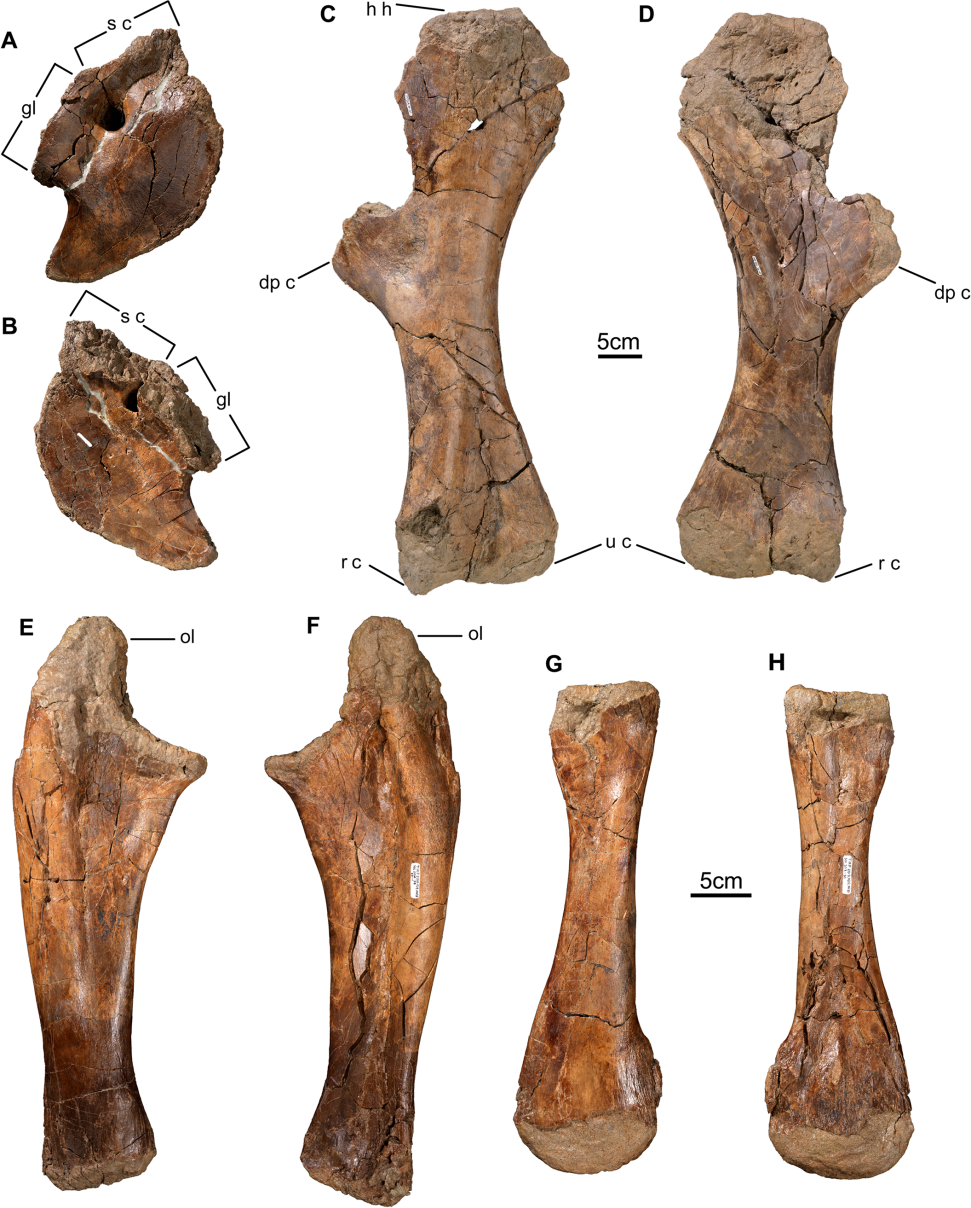
Pectoral girdle and forelimb bones of *Wendiceratops pinhornensis* gen. et sp. nov. TMP 2013.020.0049, right coracoid, in A) lateral and B) medial, views. TMP 2014.029.0100 and TMP 2014.029.0101, left humerus, in C) posterior and D) anterior, views. TMP 2014.029.0058, right ulna, in E) anterior and F) posterior views. TMP 2013.020.0030, right radius, in G) anterior and H) posterior views. Abbreviations: dp c, deltopectroal crest; h h, head of humerus; gl, glenoid; ol, olecranon process; r c, radial condyle s c, scapula contact; u c, ulnar condyle.

Numerous vertebrae from the cervical, dorsal and caudal regions have been collected, most of which preserve their neural arches, but are often crushed and/or lithostatically distorted. A syncervical has not been recovered, and only a fragment of a sacrum is known. The preserved vertebrae and ribs closely resemble those in other centrosaurine ceratopsids, including *Centrosaurus* [34] and *Styracosaurus* [35], but provide little diagnostic information beyond Ceratopsidae. The forelimbs are represented by a complete coracoid (Fig 12A and 12B), an almost complete humerus (Fig 12C and 12D), two ulnae (Fig 12E and 12F) and two radii (Fig 12G and 12H). These bones closely resemble those of other centrosaurines [34, 35]. All forelimb elements are relatively large compared to other centrosaurines known from the Belly River Group of Alberta, and suggest they belong to skeletally mature individuals [34, 35].

**Fig 13:**
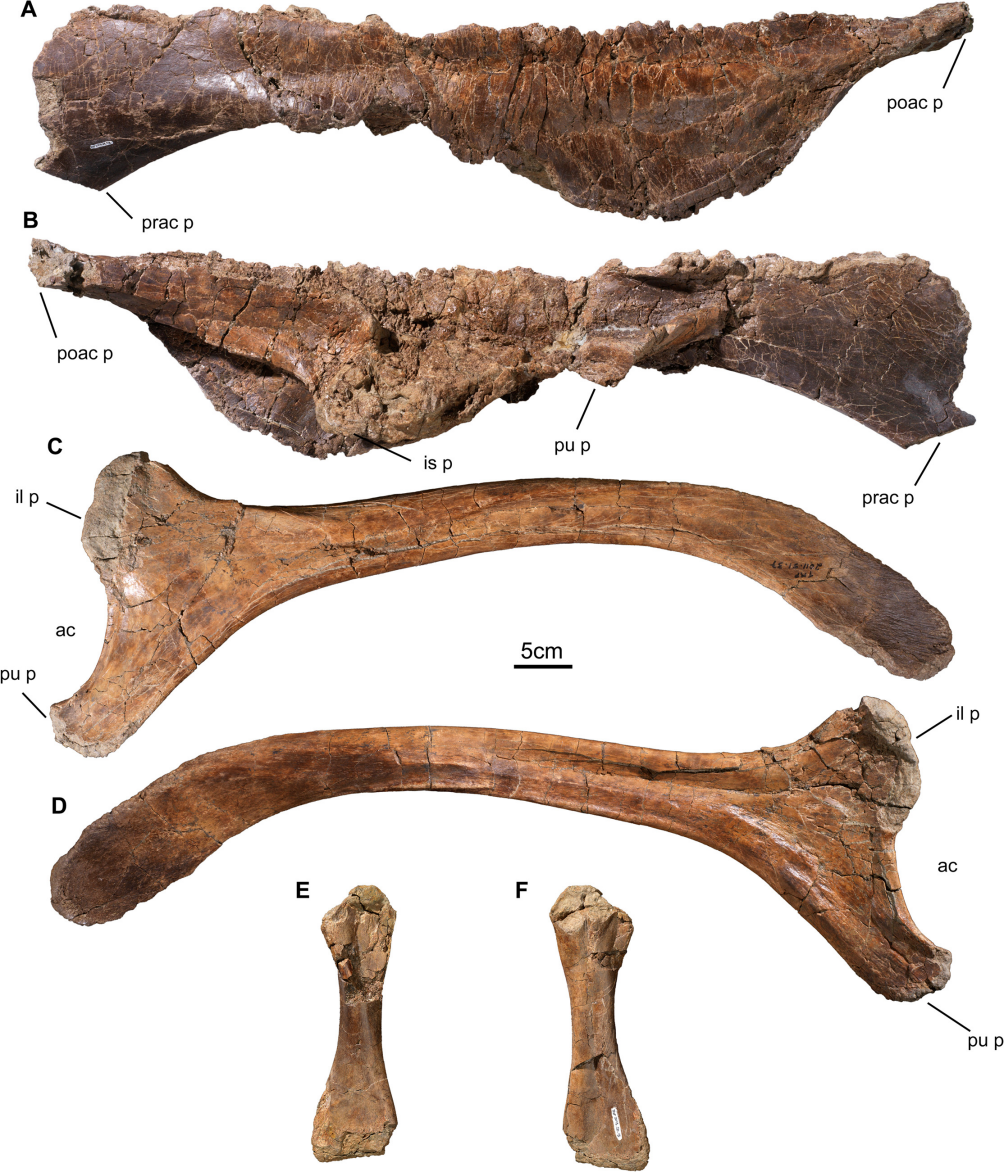
Pelvic girdle and hindlimb bones of *Wendiceratops pinhornensis* gen. et sp. nov. TMP 2014.029.0098, left ilium, in A) dorsal and B) ventral views. TMP 2011.051.0037, right ischium, in C) lateral and D) medial views. The ischial shaft thins mediolaterally and expands to form a distinctive deep, rectangular distal terminus. TMP 2013.020.0015, juvenile right tibia, in E) anterior and F) posterior, views. Abbreviations: ac, acetabulum; il p, illiac peduncle; is p, ischiac peduncle; poac p, postacetabular process; prac p; preacetabular process; pu p, public peduncle.

The pelvic girdle is represented by a largely complete, but badly fractured ilium and a complete, well-preserved ischium. The preserved ilium (TMP 2014.029.0098; Fig 13A and 13B) resembles those described for other centrosaurines, such as *Centrosaurus apertus* [34] and *Styracosaurus albertensis* [35]. The ischium (TMP 2011.051.0037; Fig 13C and 13D) is relatively thin and flat through its entire length, but this may have been accentuated by transverse crushing. The pubic peduncle is elongate and taller than mediolaterally wide throughout its length. The shorter iliac peduncle extends dorsally at a right angle from the base of the pubic peduncle, and together the pubic and iliac peduncle define the ventral and posterior margins of the open acetabulum. The shaft is relatively straight proximally and projects posteroventrally from the acetabulum. Ventral curvature of the ischial shaft occurs approximately in its midshaft region, beyond which the ischium broadens dorsoventrally and thins mediolaterally to form a deep, rectangular distal terminus in lateral view. The shape of the distal end of the ischium is distinct from other centrosaurines (e.g., *Styracosaurus* [35]; *Centrosaurus* [34]), and all other known ceratopsids, in which the distal end of the ischium tapers to a distinct blunt point in lateral view [1]. Two incomplete fibulae of large individuals and two complete tibiae of very small juveniles (total length = 200 mm) have also been recovered (Fig 13E and 13F).

#### Phylogenetic Analysis

**Fig 14:**
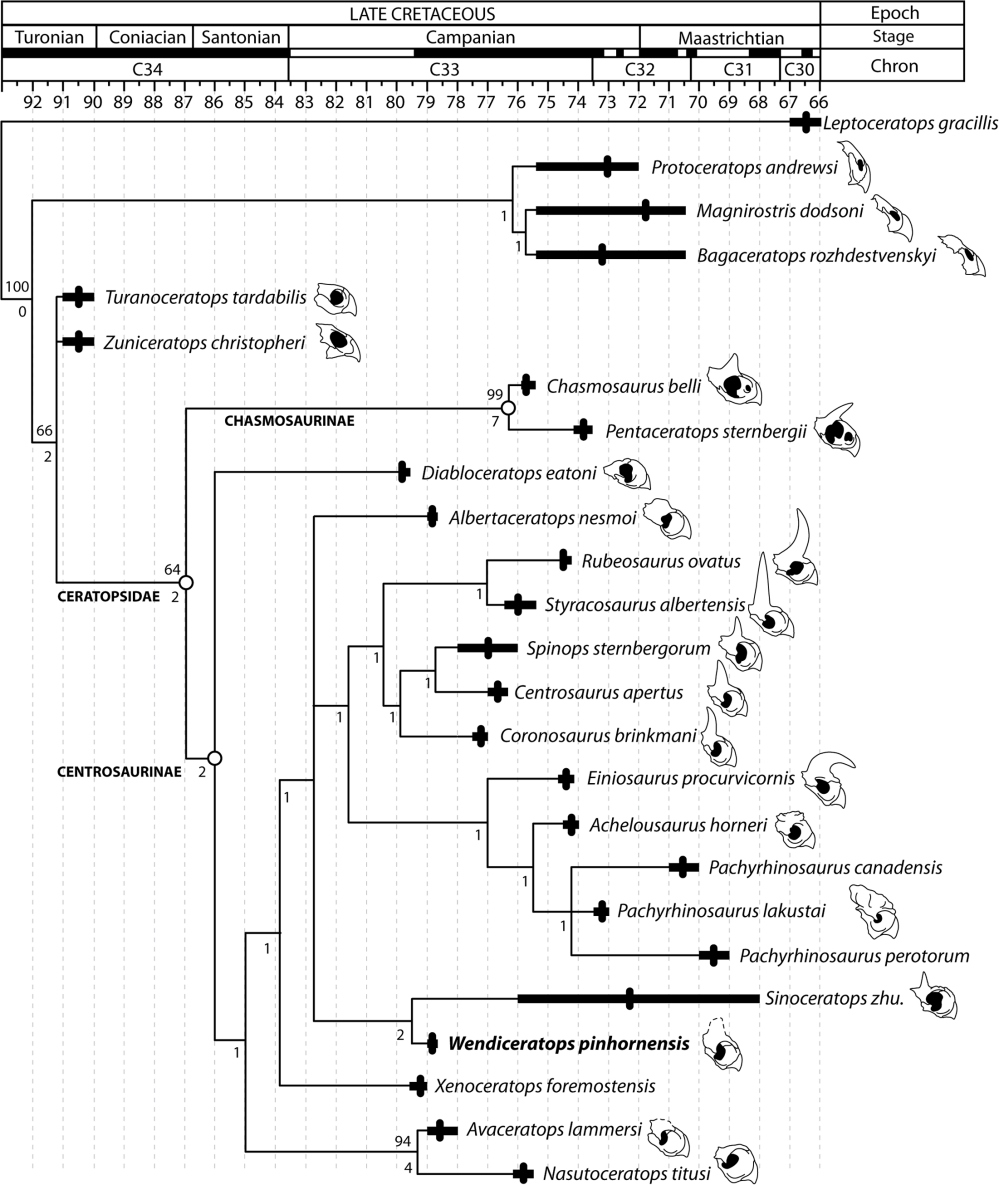
Phylogenetic relationships of *Wendiceratops pinhornensis* gen. et sp. nov. **within Ceratopsidae.** Strict consensus tree of 18 most parsimonious trees (tree length = 167; CI = 0.665; RI = 0.808) recovered in the phylogenetic analysis. See text for details. The temporal ranges of the taxa are from Sampson et al. [11], with minor modifications to the ranges of taxa from southern Alberta. Outlines to the right of taxon names represent narial morphology (rostral, premaxilla and nasal) in lateral view, and highlight the evolution of nasal ornamentation in the clade. Outlines are modified from You et al. [36] and Dodson et al. [1]. Note that ceratopsid outgroup taxa and basal centrosaurines lack well-developed nasal ornamentation.[36]

The phylogenetic analysis resulted in 18 most parsimonious trees. Each tree had a tree length of 167 steps, CI = 0.665, and RI = 0.808), with the Strict Consensus Tree showing a reasonably high degree of resolution (Fig 14). *Wendiceratops pinhornensis* is a centrosaurine ceratopsid based, in part, on the presence of a relatively wide parietal midline bar (Character 51[1]), a synapomorphy for the clade. It is recovered as the sister taxon of *Sinoceratops zhuchengensis* in all of the most parsimonious trees. The sister-taxon relationship between these two taxa is supported by four unambiguous synapomorphies: 59 (1) and 61(2), the presence of a rugose, tongue-shaped process at epiparietal locus ep 2 and ep 3 respectively, and 60 (3) and 62 (2), which refer to dorsal curvature of the epiparietals at loci ep 2 and ep 3, respectively. The relationship of the *Wendiceratops* + *Sinoceratops* clade forms a polytomy with *Albertaceratops nesmoi* and a large clade that includes *Centrosaurus apertus* and Pachyrhinosaurini.

Overall, the topology within Centrosaurinae is very similar to that recovered in the analysis in Sampson et al. [11]. *Diabloceratops* is recovered as the sister taxon to all other centrosaurines, with the *Nasutuceratops* + *Avaceratops* clade and *Albertaceratops* forming successive sister taxa to the more derived centrosaurines. However, Pachyrhinosaurini is more restricted, with *Xenoceratops* and *Sinoceratops* being recovered outside of the least inclusive clade consisting of *Centrosaurus* and *Pachyrhinosaurus* spp.

The support for any of the recovered most parsimonious topologies is weak, with Bremer decay values of one for virtually all clades within Centrosaurinae, except for the sister taxon relationships between *Wendiceratops* and *Sinoceratops* (Bremer Decay = 2) and *Nasutuceratops* and *Avaceratops* (Bremer Decay = 4), which have marginally better Bremer values. Bootstrap values are also low, with only the latter sister-pair having a bootstrap percentage of over 50% within Centrosaurinae.

## Discussion

The addition of *Wendiceratops* to the growing diversity of Ceratopsidae from the Belly River Group of Alberta contributes new information about the morphology of this clade during what appears to have been a time of rapid diversification in their early radiation [2]. The oldest named members of Ceratopsidae are the centrosaurines *Diabloceratops eatoni* from the Wahweap Formation of southern Utah and *Xenoceratops foremostensis* from the Foremost Formation of southern Alberta. These taxa are known from single localities that date from approximately 80 Ma and 79 Ma respectively. A series of taxa from the Oldman Formation of Alberta, including *Wendiceratops*, and correlative Judith River Formation of neighboring Montana, bridge the gap between the earliest known centrosaurines and the well-known succession of taxa in the Dinosaur Park, Two Medicine, and Kaiparowits formations [2]. *Wendiceratops* is the fourth centrosaurine ceratopsid recovered from the Oldman Formation, and only the second taxon recognized from the lower mudstone-dominated unit, with all of its remains being recovered from a bonebed 10 m above the contact with the Foremost Formation. *Albertaceratops* is based on an isolated skull from the same stratigraphic level (9 m above the Foremost Formation contact) collected approximately 3 km to the northeast of the *Wendiceratops* bonebed [6]. *Coronosaurus brinkmani* [8] is known from bonebeds in the overlying middle unit (Comrey sandstone zone) of the Oldman Formation in Dinosaur Provincial Park and at the Milk River Ridge reservoir, west of Warner, Alberta. *Centrosaurus apertus* occurs in the stratigraphically highest informal subdivision of the Oldman Formation, the upper muddy unit, in southeastern Alberta, where the beds are chronostratigraphically equivalent to the lower portion of the Dinosaur Park Formation in Dinosaur Provincial Park [37].

*Wendiceratop*s was essentially contemporaneous with *Albertaceratops* during the deposition of the lower unit of the Oldman Formation in Alberta, which is lithostratigraphically and chronostratigraphically equivalent to the lower Judith River Formation strata exposed in Kennedy Coulee, Montana, which are dated at approximately 78.8–79 million years old [17]. *Judiceratops tigris* and *Medusaceratops lokii*, as well as putative *Albertaceratops* material, are known from the Kennedy Coulee sequence and are therefore also approximately coeval with *Wendiceratops* in Alberta. The relatively unadorned centrosaurine *Avaceratops lammersi* is also known from the lowermost strata of the Judith River Formation [38]. The recognition of *Wendiceratops* affirms a high diversity of ceratopsids in the lower, regressive phase of the Belly River Group and correlative Judith River Formation strata, and suggests high faunal turnover rates of ceratopsid taxa coupled with some degree of niche partitioning during this time. High diversity, ecological specialization, and high faunal turnover has been documented in younger late Campanian ornithischian faunas of this region recovered from the Dinosaur Park Formation and Two Medicine formations [37, 39–42]

**Fig 15:**
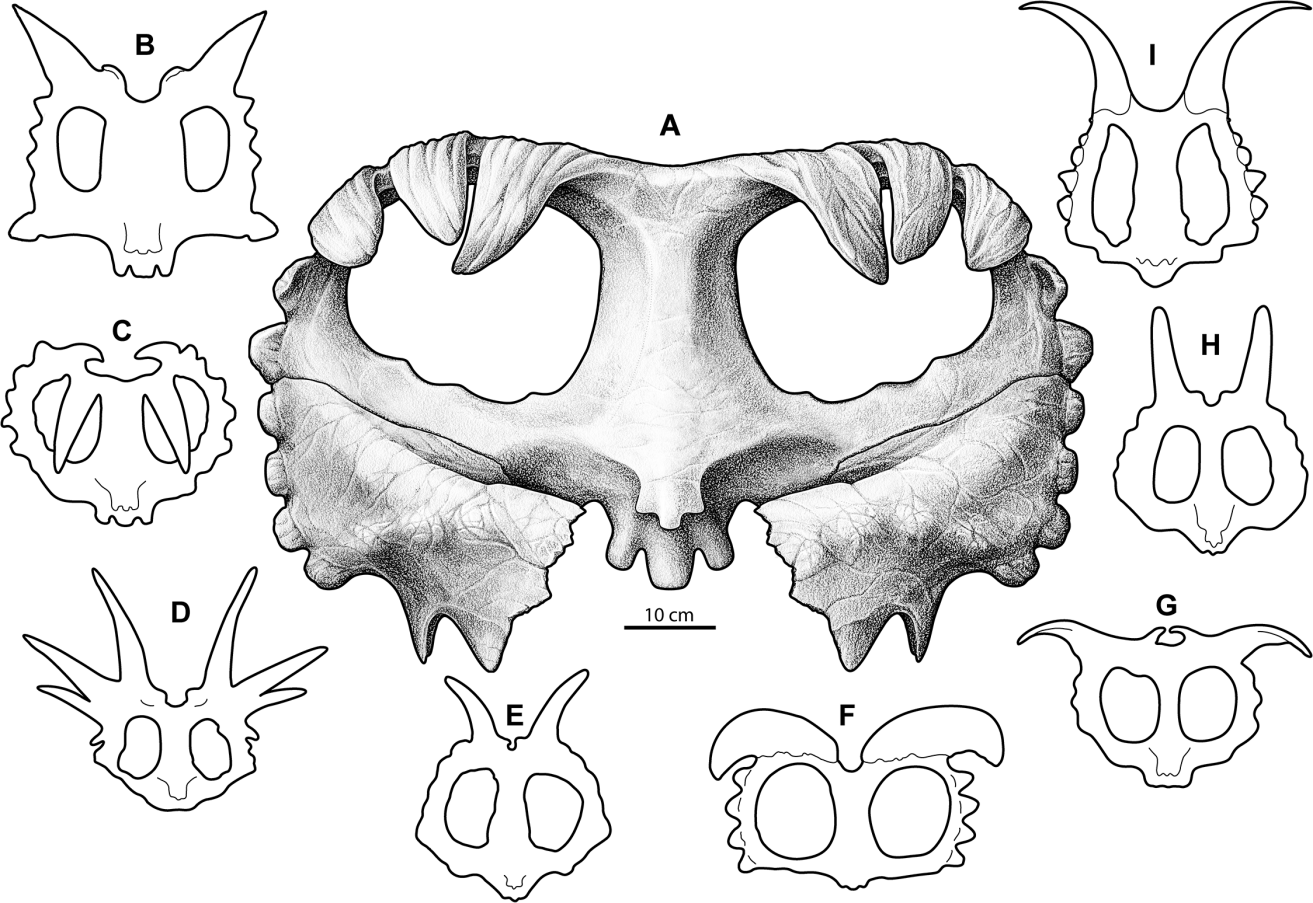
Comparative reconstructions of centrosaurine ceratopsid parietals. A, *Wendiceratops pinhornensis* gen. et sp. nov.; B, *Xenoceratops foremostensis*; C, *Centrosaurus apertus*; D, *Styracosaurus albertensis*; E, *Achelousaurus horneri*; F, *Albertaceratops nesmoi*; G, *Pachyrhinosaurus lakustai*; H, *Einiosaurus procurvicornus*; I, *Diabloceratops eatoni*.

**Fig 16:**
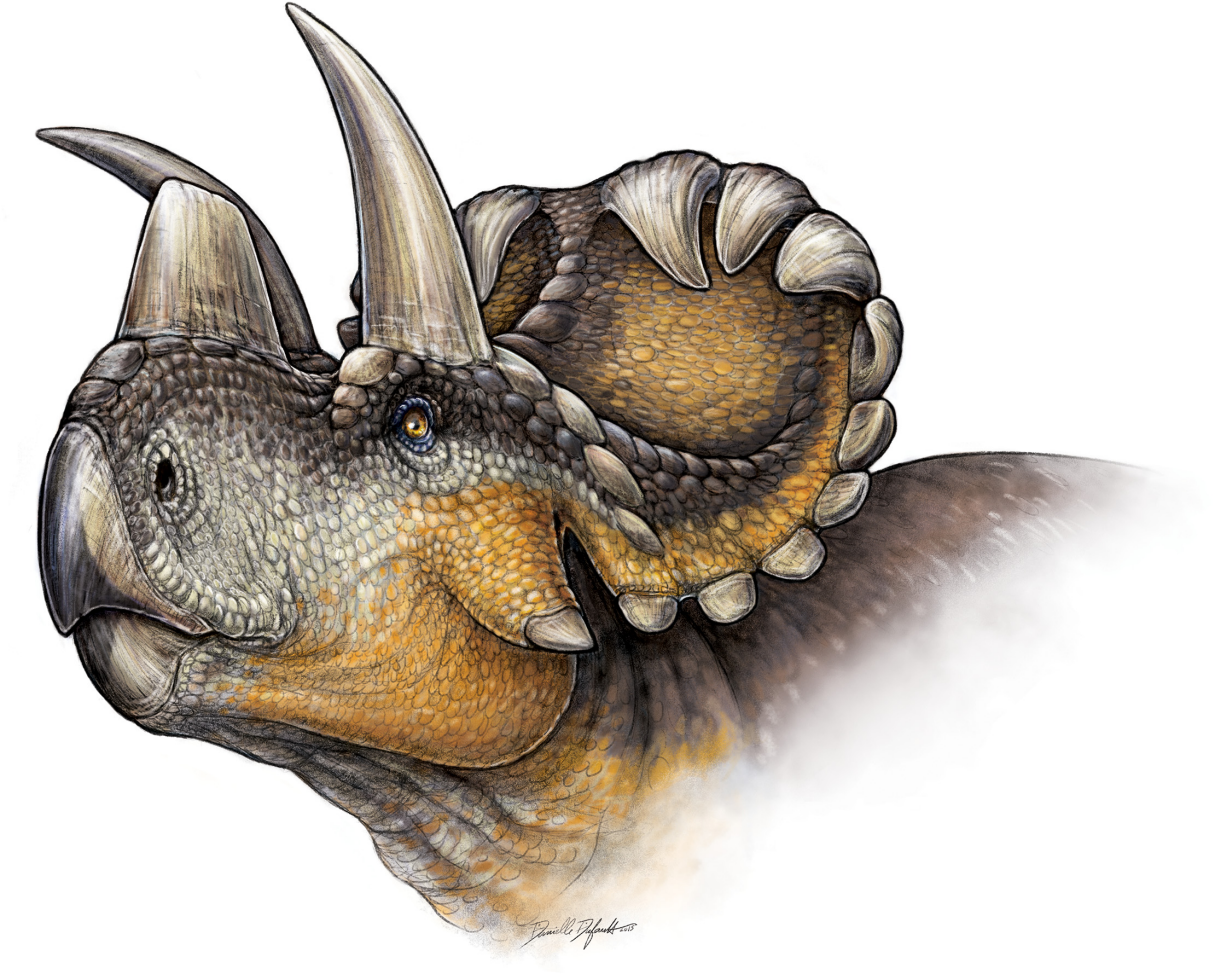
Life reconstruction of *Wendiceratops pinhornensis* gen. et sp. nov. Illustration by Danielle Dufault.

The well-developed procurved ornamentation of the parietosquamosal frill sets *Wendiceratops pinhornensis* apart from all known centrosaurine ceratopsid taxa (Figs 15 and 16). Although *Wendiceratops* shares a prominent procurved ep 1 process with numerous centrosaurine taxa (e.g., *Centrosaurus*, *Coronosaurus*, *Xenoceratops*), it is apomorphic in expressing the remaining epiparietals as strongly procurved processes. *Wendiceratops* is posited as the sister taxon to *Sinoceratops* from China, which occurs in? Campanian strata of the Upper Cretaceous Wangshi Group of Zhucheng, Shandong Province, China [30, 43]. Although the medially positioned epiparietals of *Sinoceratops* also have a dorsal inflection, as do the apices of some of the laterally positioned epiparietals of *Albertaceratops*, their curvature is not nearly as well developed as in *Wendiceratops*. *Wendiceratops* can be further differentiated from *Sinoceratops*, as well as the basal centrosaurines *Nasutuceratops* and *Avaceratops*, by the lack of the epiparietal that occupies the posterior midline margin (ep 0). *Wendiceratops* also lacks the unusual series of dorsal eminences on the posterior parietal that are apomorphic of *Sinoceratops* [30].

The series of numerous strongly procurving epiparietals overhanging the parietal fenestrae superficially resembles that seen in the chasmosaurines *Vagaceratops* and *Kosmoceratops*, but rather than coalescing along their adjacent margins, the epiparietals of *Wendiceratops* are separated by a distinctive saddle-shaped margin that typically separates large epiparietals in centrosaurines. In addition, *Wendiceratops* is also the only ceratopsid that has large, strongly dorsally reflected episquamosals. Many centrosaurines have squamosals with concave posterior bodies that result in episquamosals with apecies that are reflected away from the body. However, the episquamosal shape of the new taxon uniquely extends the distinctive parietal ornamentation along the entirety of the parietosquamosal margin.

The postcranial skeleton of Ceratopsidae typically exhibits a conserved morphology, with few characteristics differentiating individual species or even larger clades [1, 44]. The unusual shape of the distal ischium in *Wendiceratops* is, therefore, potentially significant taxonomically. In lateral view, the ischial shaft of ceratopsids reaches its maximum depth in its decurved central region, and tapers to a small point distally. This morphology is exemplified by the centrosaurines *Styracosaurus* [35] and *Centrosaurus* [34], and contrasts with the dorsoventrally broad, rectangular distal end of the ischium in TMP 2011.051.0037. Unfortunately, a complete ischium is unknown in the basal centrosaurines *Diabloceratops*, *Albertaceratops*, *Nasutuceratops*, and *Xenoceratops*, but the posterior shaft is strap-shaped and not significantly expanded distally in most non-ceratopsid neoceratopsians [36]. The broad, rectangular distal end of the ischium is, therefore, considered to represent a postcranial autapomorphy of *Wendiceratops pinhornensis*.

Although the nasal is represented by fragmentary specimens, it is inferred that *Wendiceratops* had a large, upright nasal horn, although its precise morphology remains unknown. This represents the earliest documented occurrence of a prominent nasal horn in Ceratopsia, which is otherwise well-documented only in specimens of *Coronosaurus brinkmani* [8] from the middle unit of the Oldman Formation and younger centrosaurine taxa. The most basal centrosaurines, including *Nasutuceratops*, *Diabloceratops* and *Albertaceratops* have only weakly developed nasal ornamentation comprised of a rugose ridge or small eminences above the posterior region of the nares. The presence of a large, upright nasal horn appears to be a derived character shared with *Sinoceratops* and more derived North American centrosaurines (Fig 14). However, the position of the prefrontal contact surface of TMP 2013.020.0028 relative to the position of the preserved nasal ornamentation suggests that the nasal ornamentation of *Wendiceratops* was positioned closer to the orbits than in its sister taxon *Sinoceratops*, as well as more derived centrosaurines such as *Centrosaurus* and *Styracosaurus*. The close proximity of the nasal ornamentation to the orbits is seen in other basal centrosaurines (e.g., *Nasutuceratops*, *Diabloceratops* and *Albertaceratops*), and may be plesiomorphic for Centrosaurinae. Therefore, given the intermediate phylogenetic and stratigraphic position of *Wendiceratops*, its nasal ornamentation may represent a transitional morphology between the elongate, low nasal ornamentation of basal centrosaurines (i.e., *Diabloceratops*, *Nasutuceratops*, and *Albertaceratops*) and the tall, erect nasal horncores of more derived centrosaurines such as *Coronosaurus*, *Centrosaurus*, and *Styracosaurus* (Fig 14). The anteriorly positioned, diminutive nasal horn of *Sinoceratops* may also represent a transitional morphology, or be independently derived. The nasal horn is subsequently modified within pachyrhinosaurins to a thickened, rugose nasal boss in pachyrostrans [45]. Interestingly, the transition sequence of nasal horn evolution is partially recapitulated in the ontogeny of *Pachyrhinosaurus* and presumably other pachyrostrans, in which the nasal horn is first developed as a simple undulating ridge, which transforms into a relatively taller, yet broad-based horn-like structure that is subsequently remodeled into a low, rugose nasal boss [33].

When put into a broader phylogenetic context, the detailed knowledge of nasal evolution in Centrosaurinae allows for a more complete hypothesis of nasal ornamentation evolution within Ceratopsidae. Proximate ceratopsid outgroups, including *Zuniceratops* [46], lack significant development of nasal ornamentation. All known Campanian chasmosaurines have prominent nasal horncores (where preserved), but a succession of the most basal centrosaurine taxa including *Diabloceratops*, *Nasutuceratops*, and *Albertaceratops* have relatively poorly developed nasal ornamentation. Given the condition in proximate ceratopsid outgroup taxa, and the fact that numerous basal centrosaurines lack a prominent nasal horncore, the distinctive conical nasal horns of ceratopsids likely evolved twice within the clade, and convergently in Chasmosaurinae and Centrosaurinae.

## Supporting Information

S1 File Database of specimens collected from the South Side Ceratopsian bonebed. (XLSX)

S2 File Character list used in the phylogenetic analysis of Centrosaurinae. (DOC)

S3 File Data matrix of taxa and character codings used in the phylogenetic analysis. (DOC)
