# Supplementary material for: Cranial Anatomy of Wendiceratops pinhornensis gen. et sp. nov., a Centrosaurine Ceratopsid (Dinosauria: Ornithischia) from the Oldman Formation (Campanian), Alberta, Canada, and the Evolution of Ceratopsid Nasal Ornamentation
Source: PLoS One. 2015 Jul 8;10(7):e0130007. doi: 10.1371/journal.pone.0130007 (PMC4496092; doi:10.1371/journal.pone.0130007)
Supplement: S2 File — (DOC) [file pone.0130007.s002.doc]

**S2. List of characters used in the phylogenetic analysis of Centrosaurinae. Based on Sampson et al. (2013).**

**Dermal skull roof**

1. Rostral, extent of dorsal and ventral processes: (0) triangular in lateral view, with short dorsal and ventral processes; (1) elongate, with deeply concave caudal margin and hypertrophied dorsal and ventral processes.

2. Premaxillary septum, shape: (0) rostrally elongate; (1) semicircular.

3. Premaxillary septum, nasal contribution: (0) septum formed by premaxilla only; (1) septum formed by premaxilla and nasal.

4. Premaxilla, narial strut: (0) absent; (1) present.

5. Premaxilla, septal fossa: (0) absent; (1) present.

6. Premaxilla, triangular process: (0) absent; (1) present.

7. Premaxilla, recess along ventral portion of septum: (0) absent; (1) present.

8. Premaxilla, caudoventral expansion of oral margin: (0) absent; (1) present.

9. Premaxilla, ventral extent of caudoventral oral margin: (0) at or above level of alveolar margin of maxilla; (1) well below alveolar margin of maxilla.

10. Caudoventral oral margin, composition of ventral angle: (0) premaxilla and maxilla; (1) premaxilla only.

11. Premaxilla, position of caudal tip of caudoventral process: (0) inserts into an embayment in the nasal; (1) intervenes between nasal and maxilla.

12. Premaxilla, distal end of caudoventral process forked: (0) absent; (1) present.

13. Premaxilla-nasal contact in dorsal view: (0) premaxillae insert between nasal; (1) nasals insert between premaxillae.

14. Accessory antorbital fenestra: (0) present; (1) absent.

15. Accessory antorbital fenestra size: (0) pronounced, penetration of nasal cavity visible in lateral view; (1) slight penetration, nasal cavity not visible in lateral view.

16. External antorbital fossa, size: (0) large, 20% or more length of body of maxilla; (1) greatly reduced or absent, less than 10% length of body of maxilla.

17. Maxillary tooth row, position: (0) ventrally displaced from rostral edentulous portion of maxilla; (1) at same level as rostral edentulous portion of maxilla; (Sampson et al. 2010, character 24).

18. Maxilla, maxillary cavity: (0) absent; (1) present.

19. Ectopterygoid/pterygoid complex: (0) covers entire dorsal surface and laps onto lateral surface of caudal ramus of maxilla; (1) ectopterygoid vestigial.

20. Nasal, ornamentation type in adult (ORDERED): (0) non-pronounced; (1) distinct horncore; (2) pachyostotic boss.

21. Epinasal ossification on nasal: (0) absent; (1) present.

22. Nasal, narial spine: (0) absent; (1) present.

23. Postorbital, extent of cornual sinuses in base of supraorbital ornamentation: (0) sinus invades frontal and parietal; (1) sinus enters postorbital

24. Postorbital, type of supraorbital ornamentation in subadult: (0) pointed apex, horncore at least as tall as rostrocaudally long; (1) rounded apex, horncore rostrocaudally longer than tall.

25. Postorbital, type of supraorbital ornamentation in adult: (0) horncore absent; (1) horncore; (2) rugose boss; **(modified)**.

26. Postorbital, position of supraorbital horncore: (0) centered rostrodorsal or dorsal to orbit, narrow base with caudal margin of supraorbital horncore extending to or only slightly behind caudal margin of orbit; (1) centered caudodorsal to orbit, broad base with caudal margin of supraorbital horncore extending well behind caudal orbit.

27. Postorbital, orientation of supraorbital horncore base: (0) dorsally directed; (1) dorsolaterally directed.

28. Postorbital, length of supraorbital horncore: (0) short, less than 15% basal skull length; (1) present, elongate, greater than 35% basal skull length.

29. Postorbital, curvature of supraorbital horncore in lateral view: (0) caudally recurved; (1) rostrally curved; (2) straight.

30. Postorbital, curvature of supraorbital horncore in rostral view: (0) medially recurved; (1) laterally curved; (2) straight.

31. Prefrontal-prefrontal contact: (0) absent; (1) present.

32. Palpebral, shape: (0) rod-like, articulates with prefrontal only at its base and projects across dorsal orbit, ligamentous attachment; (1) blocky, fully fused into dorsal orbital margin, sutural articulation with prefrontal and frontal.

33. Palpebral, antorbital buttress: (0) absent; (1) present.

34. Palpebral, extent of antorbital buttress: (0) present along only rostrodorsal portion of orbit; (1) present along entire rostral portion of orbit.

35. Jugal, size and orientation of jugal body: (0) projects strongly caudoventrally, does not extend below the level of the maxillary tooth row; (1) projects nearly ventrally, elongated to extend below the level of the maxillary tooth row.

36. Jugal infratemporal process: (0) absent; (1) present, contacts or nearly contacts infratemporal process of squamosal.

37. Epijugal attachment scar: (0) large blade like triangle with obtuse angle oriented towards quadratojugal; (1) scar roughly equilateral in shape.

38. Frontal fontanelle leading into supracranial cavity complex: (0) absent; (1) present.

39. Frontal fontanelle, shape: (0) transversely narrow, slit-like; (1) key-hole shaped, circular or elongate oval.

40. Parietal, rostral extent on dorsum of skull relative to occipital condyle: (0) rostral end of parietal located well in front of occipital condyle; (1) rostral end of parietal lies directly over occipital condyle.

41. Squamosal, shape of expanded blade: (0) sub-rectangular in outline; (1) triangular in outline, caudally narrowed.

42. Squamosal, rostromedial lamina forming the caudolateral floor of dorsotemporal fenestra: (0) absent; (1) present.

43. Squamosal-quadrate contact: (0) socket-like cotylus on ventrolateral squamosal for ball-like quadrate head; (1) elongate groove on medial surface of squamosal to receive lamina of quadrate.

44. Squamosal, thickened, rounded swelling along medial margin: (0) absent, lateral surface of squamosal flat to slightly convex; (1) present, lateral surface of squamosal slightly concave.

45. Parietosquamosal contact, shape in lateral view: (0) straight; (1) curved, medially concave.

46. Parietal, concave median embayment on caudal margin: (0) absent; (1) present.

47. Parietal, shape of concave median embayment: (0) shallow, restricted to center of margin; (1) shallow, entire transverse bar is a V-shaped embayment.

48. Parietal, rim on medial margin of dorsotemporal fenestra: (0) absent; (1) present, well-defined, laterally projecting rim defines medial margin of fenestra.

49. Parietal, sharp median crest: (0) present; (1) absent.

50. Parietal, rostrocaudal thickness of transverse bar at narrowest point: (0) narrow and strap-like, less than 10% total parietal length; (1) broad, 20% or more of total parietal length.

51. Parietal, median bar, transverse width: (0) narrow and strap-like, transverse width less than 10% total parietal length; (1) relatively wide, transverse width 15% or more of total parietal length.

52. Parietosquamosal frill, imbrication of marginal undulations: (0) absent; (1) present.

Epiossifications

53. Marginal dermal ossifications on parietal and squamosal: (0) absent; (1) present.

54. Episquamosal, midlateral, shape: (0) crescentic or ellipsoidal; (1) triangular or elongate.

55. Episquamosals, number per side: (0) three to five; (1) six or more.

56. Marginal ossification crossing squamosal-parietal contact: (0) absent; (1) present.

57. Epiparietals, number per side: (0) three; (1) five or more.

58. Epiparietal locus P1: (0) absent; (1) present.

59. Epiparietal, shape of locus P2: (0) low D-shaped process, wider than long; (1) rugose tongue-shaped process, less than twice as long as wide (2) elongate flattened process or spike, greater than twice as long as wide; **(modified)**.

60. Epiparietal, curvature of locus P2: (0) straight; (1) laterally curved; (2) medially curved; (3) dorsally curved; (Sampson 1995, character 15, modified).

61. Epiparietal, shape of locus P3: (0) low D-shaped or triangular process; (1) rugose tongue-shaped process, less than twice as long as wide (2) elongate flattened process or spike, greater than twice as long as wide; **(modified)**.

62. Epiparietal, curvature of locus P3: (0) uncurved or slightly medially curved; (1) laterally curved; (2) dorsally curved (new character).

63. Epiparietal, locus P4 shape: (0) low raised D-shaped process; (1) elongate spike; (Sampson et al. 2010, character 102, modified).

64. Epiparietal, locus P5: (0) absent; (1) present; (new character).

65. Epiparietal, locus P5 shape: (0) low D-shaped or triangular process; (1) elongate spike; (new character).

66. Epiparietal, locus P6: (0) absent; (1) present; (new character).

67. Epiparietal, locus P6 shape: (0) low D-shaped or triangular process; (1) elongate spike; (new character).

68. Epiparietal, locus P7: (0) absent; (1) present; (new character).

**Braincase**

69. Supraoccipital, contribution to foramen magnum: (0) forms dorsal margin of foramen magnum; (1) eliminated from margin by exoccipital-exoccipital contact on midline; (Forster 1990, character 63).

**Lower jaw**

70. Predentary, dentary processes: (0) ventral processes much longer than abbreviated dorsal processes; (1) dorsal and ventral processes elongate and subequal in length; (Sampson et al. 2010, character 114).

71. Predentary, orientation of triturating surface: (0) nearly horizontal; (1) inclined steeply laterally; (Dodson et al. 2004, character 57).

72. Dentary lateral ridge confluent with cutting surface of predentary: (0) present; (1) absent; (Sampson et al. 2010, character 116).

73. Dentary, caudal extent of tooth row: (0) terminates at the center of the coronoid process; (1) terminates caudal to the coronoid process; (Chinnery and Weishampel 1998, character 18).

Dentition

74. Teeth, number of roots: (0) one; (1) two; (Forster 1990, character 34).

75. Teeth, number of alveoli in dentary: (0) fewer than 20; (1) more than 20; (Makovicky and Norell 2006, character 104).

76. Teeth, number of replacements per alveolus: (0) one or two replacement teeth; (1) three or more replacement teeth; (Sereno 1999, character 137).

Axial skeleton

77. Cervical vertebrae, formation of syncervical: (0) C1-3 fused or tightly articulated, atlantal hypocentrum present as a ventrally placed, wedge-like bone; (1) C1-3 firmly fused, atlantal hypocentrum forms a complete ring; (Forster 1990, character 122)

78. Axis, neural spine shape and orientation: (0) blade-like and nearly vertical, overhangs only rostralmost portion of C3; (1) blade-like morphology lost, spine steeply angled to reach caudal margin of C3; (Sereno 1999, character 141)

79. Atlantal rib: (0) present; (1) absent; (Sampson et al. 2010, character 129).

80. Dorsal vertebrae, shape of centra: (0) relatively axially elongate; (1) axially shortened; (Sampson et al. 2010, character 130).

81. Sacrum, longitudinal sulcus on ventral surface: (0) absent; (1) present (Sereno 1999, character 144).

**Pectoral girdle and forelimb**

82. Scapula, relative contribution to glenoid fossa: (0) scapula and coracoid contribute equally; (1) scapula contributes well over half of the glenoid; (Sereno 1999, character 145).

83. Olecranon process: (0) relatively small; (1) enlarged (>one-third of ulnar length); (Forster 1990, character 104, modified).

84. Clavicle: (0) present; (1) absent; (Sereno 1999, character 147).

85. Manual and pedal unguals, shape: (0) taper to distal tip; (1) dorsoventrally flattened with blunt and rounded distal tips; (Chinnery and Weishampel 1998, character 64).

86. Manual and pedal penultimate phalanges, shape: (0) length exceeds width; (1) width exceeds length; (Sampson et at. 2010, character 137).

**Pelvic girdle and hind limb**

87. Ilium, lateral eversion of dorsal margin: (0) absent; (1) present; (Forster 1990, characters 108-109, modified).

88. Ilium, relative lengths of pubic and ischial peduncles: (0) pubic and ischial peduncles long, extend well below body of ilium approximately the same distance; (1) ischial peduncle reduced along ventral aspect, pubic peduncle projects further ventrally than ischial peduncle; (Sampson et al. 2010, character 139).

89. Pubis, prepubic process: (0) short and unexpanded distally; (1) elongate, distal end greatly expanded dorsoventrally; (Forster, 1990, character 111).

90. Pubis, position and length of postpubic rod: (0) relatively short but extends past ischial peduncle of ilium, arises ventral to acetabulum and lies along ventral and ventromedial margin of ischium; (1) very abbreviated, terminates at level of ischial peduncle, arises medial to acetabulum and passes entirely medial to ischium; (Forster, 1990, character 110).

91. Pubis and ischium, morphology of contributions to acetabulum: (0) pubic acetabular surface faces caudolaterally, pubis and pubic process of ischium contribute equally to ventral margin of acetabulum; (1) pubic acetabular surface faces laterally and forms a partial medial wall to the acetabulum, pubic process of ischium elongate and meets pubis close to anterior margin of acetabulum, ventral portion of pubic acetabular surface lies medial to pubic ramus of ischium; (Sampson et al. 2010, character 142).

92. Ischium, cross-sectional shape of shaft: (0) thick and ovoid; (1) laterally compressed and bladelike, tapered dorsally; (Forster 1990, character 112).

93. Ischium, orientation of shaft: (0) nearly straight or slightly decurved; (1) broadly and continuously curved; (Forster 1990, character 113).

94. Femur, morphology of greater and lesser trochanters: (0) trochanters distinct and located below the level of the femoral head; (1) trochanters coalesced and level with the femoral head; (Dodson et al. 2004, character 72).

95. Femur, size of fourth trochanter: (0) large and pendant; (1) small, reduced to low prominence; (Sereno 1999, character 154).

96. Femur-tibia proportion: (0) tibia longer than femur; (1) femur longer than tibia; (Forster 1990, character 103).

97. Pes, metatarsal proportions: (0) length of MT I two-thirds the length of MT II; (1) MT I reduced to one half or less the length of MT II; (Sampson et al. 2010, character 148).

98. Dorsal squamosal ridge: (0) absent; (1), weakly developed; (2) prominent series of bumps or continuous raised ridge; **(new character).**

99. Epiparietal, locus P0: (0) absent; (1) present; **(new character).**

100. Epiparietal, shape of locus P1: (0) low D-shaped process, wider than long; (1) rugose tongue-shaped process, less than twice as long as wide (2) elongate flattened process or spike, greater than twice as long as wide; **(new character).**

101. Epiparietal, curvature of locus P1: (0) straight; (1) laterally curved; (2) dorsally curved; **(new character).**
