# Supplementary material for: Cranial Anatomy of Wendiceratops pinhornensis gen. et sp. nov., a Centrosaurine Ceratopsid (Dinosauria: Ornithischia) from the Oldman Formation (Campanian), Alberta, Canada, and the Evolution of Ceratopsid Nasal Ornamentation
Source: PLoS One. 2015 Jul 8;10(7):e0130007. doi: 10.1371/journal.pone.0130007 (PMC4496092; doi:10.1371/journal.pone.0130007)
Supplement: S3 File — (DOC) [file pone.0130007.s003.doc]

**S3. Data Matrix used in phylogenetic analysis based on characters listed in S2.**

***Leptoceratops gracilis*** 1??0???0010001?00?0??0?00?????000?0000?0000000?00????????0??????????0000000000000000000000000000000??

***Protoceratops andrewsi*** 0??0???000000010000000?00?????000?0000?0010000?0000?0????0??????????0000000000000000000000000000010??

***Magnirostris dodsoni*** 1??0???000110000000100??10002?000?0000??????0???????????????????????00100?0??????????????????????????

***Bagaceratops rozhdestvenskyi***  1??0???000100000000000??0?????000?0000?001??00??0???????????????????0010000???????????????????????0??

***Turanoceratops tardabilis*** ???????????????0000?????1001?1?1?????0???????????????????????????????00????0?????????????????????????

***Zuniceratops christopheri*** 1000000???0100000000?100100102?1101100??0?0????0?10?0???????????????00000000???110?????????01?0??????

***Chasmosaurus belli*** 100111000?0100111111101?100(0&1)0201111111111011111110001110010302?0?0?0110111111111111111111111111111002

***Pentaceratops sternbergii*** 100111000?0110111111101010111201111111111011111110001110010000?0?0?01101111111111111111111111?1111002

***Diabloceratops eatoni*** 01100001010010000?0001??100102?11011010001100101111010011100000101001??????1?????????????????????1021

***Albertaceratops nesmoi*** ?1?000??????01?101?00???10?112?111111??001100101111110011100000100?0?1101111?????????????????????1021

***Rubeosaurus ovatus*** 01??0001?1??0??????1??0?1000???1?????????????101??11100?1000201111?1??????????????????????????????0??

***Styracosaurus albertensis*** 01100001111001?1111101001000??1111111100011001011111100111(0&1)(0&2)21111111111011111?111101111111101111?1012

***Spinops sternbergorum*** ??1?00???????1?????1??0?1000???111???10??????101111?100??120?????????????????????????????????????1022

***Centrosaurus apertus*** 01100001111001?11111010010002211111111000110010111111001111200010101111011111111110111111110111111022

***Coronosaurus brinkmani*** 011?000111??01?11111010010102111111111000110010111111001111011010101111011111?11?1????111110?111?1012

***Xenoceratops foremostensis*** ????????????????????????1??1????????????0110010?11101?011120???1?1????????????????????????????????012

***Sinoceratops zhuchengensis*** ??1??????????001???1????0?????010????100011000?111111001111312010????????????????1????11?????11??1112

***Einiosaurus procurvicornis*** ?11?000??????1?1111101112000??010?1111000110010111111001100220010101111011111?11?1??1?111??1111??10??

***Achelousaurus horneri*** 011?00011110?1?1111201112000???10?1?1100011001011111100?10022101010111101111?????????????????????10??

***Pachyrhinosaurus canadensis*** 011000011110?1?11112011?2100???10?111100?1100??11??11???101221010101111011?????1??????????????1??10??

***Pachyrhinosaurus lakustai*** 01100001111001?1111201112100???10?1?11000110010111111001101221010101111011111?11?1??11111110111??10??

***Pachyrhinosaurus perotorum*** 01?0000111???1?11?1201??2100???10????1????1?010?111?1???11????0??????????1?1???1???????????0111???012

***Avaceratops lammersi*** 01?000011110?1?10100011?101111?11011?100011000?11??0100?110000010?011??011111111?10?11???????1?1?2100

***Nasutoceratops titusi*** 01100001111001?10100011?101110?110??1100011000?111101001110000010101???????111?1?10??????????????2100

***Wendiceratops pinhornensis*** ?????????????1?101?10???????????????????0110010?111110011113120100?0?????11????1?10?1?11???11????2012
